# Multilayer Game Collaborative Optimization Based on Elman Neural Network System Diagnosis in Shared Manufacturing Mode

**DOI:** 10.1155/2022/6135970

**Published:** 2022-09-23

**Authors:** Qi Tang, Baotong Wu

**Affiliations:** School of Management, Shenyang University of Technology, Shenyang 110870, China

## Abstract

With the dynamic sharing of global resources shifted from the independent production of a single enterprise to and the development of collaborative manufacturing, today's manufacturing industry has collaborative production among multiple enterprises. This paper extends the optimization method of the multienterprise dynamic equipment collaborative scheduling system with the dynamic manufacturing network theory approach, combining the neural network system and model predictive control theory to continuously and dynamically diagnose the changes of demand and capacity in the actual production process and design the corresponding multilayer game collaborative optimization robust model according to the uncertainty relationship. The study considers the production characteristics and management needs of multiple enterprises within the manufacturing industry, as well as the optimization of the dynamic equipment collaborative scheduling system, uncertain demand, dynamic equipment matching effectiveness, and equipment failure under the shared manufacturing model, deduces the market capacity demand for manufacturing enterprises by inverse prediction of dynamic market demand through the simple recurrent neural network (Elman network), and then maximizes the idle equipment utilization and load capacity under the shared manufacturing model in model rolling optimization. This paper gives model solutions based on big data analysis and combines improved population intelligence algorithms to determine the dynamic matching of manufacturing resources, product supply and demand paths, and transportation flows. The simulation results show that the application of model predictive control theory to the model of Elman neural network system diagnosis can effectively reduce the adverse effects of the two-way uncertainty environment on the profitability of enterprises. Meanwhile, considering the maximum performance matching of dynamic equipment scheduling and combining with robust optimization methods will reduce the cost loss of manufacturing enterprises in the worst case, and the cost loss reduction can reach 24.73%. The analysis results verify that the method is of great practical significance in the direction of intelligent production collaboration and industrial digital transformation.

## 1. Introduction

Economic globalization has gradually eliminated barriers between countries and regions, and factors of production have flowed freely around the world. Reasonable resource allocation has brought about major changes in the development environment of the manufacturing industry. Many manufacturing enterprises have changed their spatial layout to achieve greater economic benefits and cater to larger market demands. Negri et al. proposed that in Industry 4.0 and Smart Manufacturing research, optimization and decision-making can be achieved through digital twin (DT) technology and rely on synchronization enabled by sensors to update the same data in real-time with the physical system [1]. Villalonga et al. proposed that an important challenge for today's cyber-physical production systems is to update dynamic production schedules through automated decisions executed at production runtime [2]. This shows that in the era of Industry 4.0, the real-time feedback and monitoring of production data will help manufacturers improve their own production efficiency and productivity. We use the existing sensor monitoring system to monitor the production data in real-time, analyze the equipment operation, and analyze the equipment failure problems and the real-time data changes in production capacity to achieve a more efficient and reliable production method. And in the process, in the face of insufficient capacity, we can dynamically realize the way of equipment scheduling to improve the production capacity of enterprises. This new manufacturing concept, which breaks through spatial restrictions and realizes physical space and cyberspace, has led the previous static resource allocation to a more flexible and dynamic resource allocation. In order to improve the problems of inconvenient communication, inefficient resource utilization, and inability to complete manufacturing tasks efficiently in the manufacturing industry, resource sharing has become an important manufacturing method to promote the transformation and upgrading of the manufacturing industry [3]. In order to better meet customer needs and respond to market demand faster, more and more manufacturing companies have begun to say goodbye to the traditional manufacturing model, and collaborative manufacturing has become a new manufacturing model adopted by manufacturing companies [4]. Collaborative communication between enterprises is an important bridge to achieve information and resource sharing [5]. Zhu et al. proposed that with the support of supply chain comanufacturing platform, the overall competitiveness of supply chain comanufacturing can be effectively improved, and the real-time interaction and information sharing of material, information, and capital flow between suppliers, manufacturers, and customers can be realized [6].

Current research on collaborative manufacturing focuses on supply chain collaborative manufacturing, collaborative manufacturing systems, collaborative industrial design, and collaborative production planning. In the area of collaborative manufacturing in supply chain, Li et al. studied the business process and resource interaction mode of collaborative manufacturing in supply chain by focusing on the core production enterprises and five supply chain business links from collaborative planning, collaborative procurement, collaborative production, collaborative logistics, and collaborative service; this will enhance the overall competitiveness of collaborative manufacturing in supply chain and realize the real-time interaction and information sharing among suppliers and manufacturers and real-time interaction and information sharing of material flow, information flow, and capital flow among suppliers, manufacturers, and customers [3]. Li et al. proposed a multiobjective decision model to maximize the manufacturing matching degree in a collaborative supply chain environment and validate the feasibility and effectiveness of the method [4]. In the construction of the collaborative manufacturing system, Wang et al. analyzed the production characteristics, typical production processes, and collaborative management requirements of multiroom production in discrete manufacturing enterprises and proposed the collaborative manufacturing execution system architecture for discrete manufacturing [7]. Lin et al. discussed the conceptual design of a global decision support system for SMEs actively involved in collaborative manufacturing and proposed web service-based system architecture to provide maximum interoperability among all distributed participants of the collaborative manufacturing network and their management information systems [8]. As for collaborative industrial design, Cao et al., by analyzing the basic concept and application status of collaborative product design, pointed out that collaborative innovation is an effective way to solve the weak innovation capability of small enterprises common in industrial design; the collaboration between industrial design companies and manufacturing companies will be beneficial to the collaborative integration among industrial design industry chain members [9]. Wang et al. proposed a networked collaborative design system and established a digital assembly model independent of various commercial CAD software according to the requirements of collaborative product development [10]. In the area of collaborative production planning, Shin proposed a collaborative coordination mechanism to address the problem of self-coordination faced by manufacturing entities due to different organizational practices, constraints, and conflicting goals by studying the collaborative decision-making approach to operational coordination among manufacturing facilities in a manufacturing logistics network [11]. Cheng et al. proposed a supernetwork-based model for the scheduling problem of distributed manufacturing operations and conducted group experiments to discuss the different cases of combining distributed and collaborative manufacturing [12]. Based on the analysis of the impact of product modularity on collaborative innovation, Wang and Shu constructed a customer-driven, module-based innovation carrier and sustainable collaborative innovation network model, which provides an effective way for global manufacturing companies to manage external partners [13]. As for shared manufacturing, the current research mainly focuses on manufacturing resource service sharing, manufacturing information, and knowledge sharing. In manufacturing resource service sharing, Wang et al. proposed an integrated architecture to facilitate resource allocation in shared manufacturing in view of the difficulty of adapting traditional centralized optimization methods to cross-organizational resource coordination [14]. Du et al., after analyzing the management characteristics of the manufacturing industry, proposed an order-oriented cloud service library and an order-based shared manufacturing resource model, making it easier for heterogeneous resources in different regions to collaborate remotely [15]. Liu et al. analyzed the resource and service sharing model in traditional networked manufacturing and conducted a detailed theoretical and modeling analysis on resource service utilization and demand satisfaction rate and revealed the challenges and development directions faced nowadays [16]. In the area of manufacturing information sharing, Gao and Nee provided an overview of reported research on manufacturing-related knowledge management and focused on knowledge sharing in the product development process, highlighting manufacturing knowledge sharing methods related to the product development process and pointing out future challenges and research directions [17]. Sun et al. established a platform architecture for real-time information sharing in manufacturing supply chains to improve the efficiency of information collection and sharing based on the problem that the information collection in the current stage of manufacturing supply chain information sharing platform is traditional, which affects the speed of real-time information sharing [18]. Lopez-Ortega and Ramirez analyzed relevant information systems, servers, and prototype client applications to facilitate data sharing and exchange of flexible manufacturing resources based on manufacturing systems with relevant capabilities for data sharing and exchange [19]. Song proposed to build a collaborative networked manufacturing system based on product lifecycle manufacturing cycle and manufacturing information sharing and verified the feasibility and effectiveness of the method by simulation [20]. In terms of knowledge sharing, Wang and Wan proposed a knowledge sharing mechanism among manufacturing resources based on cloud-edge collaboration; the effectiveness of the proposed method was verified by comparing manual and knowledge sharing configurations in a comparative experiment on a multivariety, low-volume manufacturing experimental platform [21]. Zhang et al. proposed a resource-centric strategy to obtain production-related data and make decisions for the management of shared resources for resource monitoring and maintenance [22]. Zhang et al. suggested that green knowledge sharing and business model innovation are important ways for new firms to achieve performance growth and provide direction for new firms within the manufacturing industry to implement green innovation strategies to improve performance [23].

The application of the Elman neural network and swarm intelligence algorithm is an important part of realizing the optimization of the dynamic equipment scheduling system. In recent years, scholars in various industries have mentioned the importance and innovation of the neural network discipline in their own research. Based on the development of the artificial neural network and the continuous improvement of swarm intelligence algorithms, the manufacturing industry has also developed rapidly. We found that swarm intelligence algorithms are superior to traditional methods in terms of speed and quality in solving large-scale problems. Some scholars have pointed out that population intelligence algorithms have certain research prospects in the fields of autonomous driving and intelligent transportation, emergency response, and environmental monitoring and are better than traditional algorithms in data transmission in the Internet of Things (IoT) [24, 25].

The neural network is an important research field for artificial intelligence applications, which is a network composed of a large number of simple computing units (artificial neurons) interconnected. The neural network is a certain degree of abstraction, simplification, and imitation of the information processing function of the human brain nervous system on the basis of human understanding of the working mechanism of the brain. Compared with traditional intelligent algorithms, neural network models have the following characteristics: First, neural networks have nonlinear mapping capabilities; they do not require accurate mathematical models; they are good at learning and integrating effective knowledge from input and output data. In the past 20 years, neural network technology has achieved great success and progress in intelligent control, pattern recognition, computer vision, nonlinear optimization, and signal processing. Zhao and Yao proposed a novel prognostic framework based on multisensory signal features and called it a multidislocation time series convolutional neural network (MDTSCNN) [26]. Cruz et al. combined an integrated convolutional neural network and an evolutionary algorithm to propose a novel image classification method [27]. Xie et al. proposed a short-term electric load forecasting method combining the Elman neural network and particle swarm optimization algorithm [28]. Fernandes Junior and Yen combined deep convolutional neural networks and particle swarm algorithms to achieve more efficient and stable image classification tasks [29]. When we build a collaborative scheduling system based on a two-way uncertainty environment, we find that this application based on the Elman neural network and improved swarm intelligence algorithm makes the system more stable and reliable. We build models by using the basic principles of neural networks to establish the link between equipment failure factors and certain common parameters in actual production processes.

The modern control theory developed and perfected in the early 1960s has the optimal performance indicators and systematic and precise theoretical design methods and has made outstanding achievements in aerospace, guidance, and other fields. Modern control theory still has room for improvement in the control of industrial processes, which will realize higher industrial value conveniently and quickly. The problem in industrial processes is that the basis of modern control theory is an accurate object parameter model, while industrial processes often have the characteristics of nonlinearity, time-varying, strong coupling, and uncertainty, and it is difficult to obtain an accurate mathematical model, so the control effect is greatly reduced. Some scholars have further optimized the model based on the evaluation process in the manufacturing mode combined with the neural network theory. Gao constructed a comprehensive performance evaluation model based on the backpropagation (BP) neural network and a rough set to improve the performance evaluation of manufacturing collaborative logistics [30]. In addition, this study obtains a set of key performance indicators by using the rough set attribute parsimony theory to screen and optimize the evaluation indicators. Then use the BP neural network to predict and evaluate the key performance data, which greatly reduces the training time and shortens the learning time. Quan and Zhang based on the background of intelligent subsidiary suppliers, used machine learning to study the evaluation method of supplier efficiency [31]. Based on the traditional backpropagation neural network, combined with the improved particle swarm optimization algorithm, this research constructs a new neural network model based on the supplier evaluation index system and evaluates the supplier efficiency of intelligent subsidiaries. Saravanan et al. proposed a new method to simulate manufacturing cost tolerance and optimize the tolerance value as well as manufacturing cost [32]. The study combined a genetic algorithm with a neural network model to optimize tolerance values. This approach will enable design and process planning engineers to make informed decisions. Wang evaluated the manufacturing capability of smart job shops based on an improved backpropagation neural network [33]. First, the study summarizes the core production factors that affect the improvement of the manufacturing capacity of the smart job shop and constructs a multilevel scale for the manufacturing capacity of the smart job shop. At the same time, based on the evaluation of job shop scheduling, this research establishes a framework for evaluating the manufacturing capability of an intelligent job shop, thereby improving the evaluation capability of the model. Shi et al. proposed a metadata replication method with a separate replication strategy, combined with the intelligent production line network platform, carried out research on a neural network-based intelligent production line network state prediction system, and designed a network [34]. The prediction system further prejudges the operation status of the intelligent production line network. Gegovska et al. analyzed the data using a multiobjective decision model with the goal of raising awareness and addressing the needs of green supplier selection [35]. Ultimately, the research achieved a better solution by incorporating artificial neural networks. Huo et al. proposed a discrete manufacturing workshop production control method based on ant colony optimization. This research describes the production management and control problems of discrete manufacturing workshops by analyzing the functions of the management and control system and then establishes the corresponding mathematical model [36]. Finally, the research solves the static multiobjective production control problem and verifies the effectiveness of the method with a numerical example. Wang built a deep learning prediction model based on a bidirectional long short-term memory network and a self-supervision mechanism and completed order management and completion date prediction [37]. Ramírez et al. developed a new fuzzy controller and applied it to the problem of nickel recovery, minimizing fuel consumption and environmental pollution [38]. Fan and Zhang set up experiments to conduct multisystem fusion of neural networks, combined with big data and artificial intelligence, to verify the efficient operation of neural networks in multisystem fusion [39]. Using the method of mathematical analysis and big data fitting, the collected data are classified to improve the comprehensive work efficiency of the system. Jiang et al. proposed an end-to-end model for the prediction of multistep machine speed [40]. The model includes a deep convolutional LSTM encoder-decoder architecture and combines examples to verify the feasibility of the method. Mawson and Hughes proposed and compared the use of two deep neural networks to predict manufacturing based on production schedules, climatic conditions, thermal characteristics of facility buildings, building behavior, and usage energy consumption patterns of the facility and plant conditions. Ultimately, the study provided a predicted building energy profile that the model can use to identify peaks in energy consumption [41]. Hu analyzed the factors affecting product design decisions based on the development of products. This research introduced the basic process of product modeling, designed a model based on image processing under the background of big data, and built a decision-making model for product modeling and designed scheme under the big data cloud environment [42]. Finally, experiments proved that the decision-making model can improve the overall design efficiency, shorten the manufacturing cycle, and provide new ideas for product modeling design. Liu and Dai constructed a quality classification model based on a data generation model and described the process of water heater lining quality classification under small sample data and unbalanced environments [43]. Beruvides et al. proposed a real-time monitoring system and implemented an adaptive neurofuzzy inference system model, resulting in high fitting accuracy and good generalization capability [44]. Wang et al. proposed a new deep learning-based machine vision inspection method for identifying and classifying defective products without loss of detection accuracy [45]. Cruz et al. combined with a convolutional neural network (CNN) proposed a structured light-based computer vision system for LPG pressure vessel welding inspection and verified the robustness of the system [46]. Xu and Zhou presented a new object detection framework, classification priority network (CPN), and a new classification network, multigroup convolutional neural network (MG-CNN), to inspect the defects of steel surface. The proposed method had yielded best performances compared with prior methods used in defect inspection [47].

With the further improvement of manufacturing capacity, new problems have emerged. The market demands faced by today's manufacturing companies have become more complex, and market demands are susceptible to fluctuations due to environmental influences. Manufacturing enterprises themselves face problems such as low equipment load and wastage of production capacity in the manufacturing process. At the same time, with the introduction of manufacturing concepts such as shared manufacturing, cloud manufacturing, and shared manufacturing, the matching method and matching degree between idle equipment and resource agents have gradually attracted the attention of scholars. Through the analysis of the existing literature, it can be found that scholars mainly focus on the matching of supply and demand and their combination with process industry production, but lack the analysis of the matching efficiency of dynamic equipment. For the prediction method of equipment failure, the traditional prediction method is difficult to reasonably design the weight according to different equipment models, and the prediction process often has a certain degree of subjectivity. For the existing swarm intelligence algorithms, it is often easy to fall into the local optimal solution, and it is difficult to achieve the maximum optimization of the scheduling system. In addition, there are still research gaps in the construction of the scheduling platform and the matching principle of dynamic equipment. Most of the existing dispatching platforms are built by uploading only the basic information of both supply and demand without deeply integrating the production capacity and failure problems of enterprises, and the corresponding matching principle exists only on the basic capacity demand without considering the uncertainties existing in the dynamic environment, resulting in a poor matching effect from time to time. This paper studies the optimization of the dynamic equipment scheduling system in shared manufacturing mode and explores how to combine the uncertain market demand and the uncertain dynamic equipment matching relationship to maximize the benefits of manufacturing enterprises themselves. In actual operation, the reasonable matching of dynamic equipment will enhance the production capacity of manufacturing enterprises and reduce unnecessary energy, labor, and material losses. In the existing literature, the product failure rate is not considered much, but the equipment capacity considered in this paper already includes the consideration of product defect rate. When the equipment causes product failure due to its own aging or operation error, this part of the capacity is waste capacity and is not calculated in the equipment's own production capacity. Therefore, the basic calculation of equipment failure rate and production capacity includes the problem of defective products.

Therefore, under the background of the above practice and research, this paper makes the following contributions: (1) We combine the concept of dynamic equipment scheduling under the shared manufacturing mode and take the differentiated needs of customers as the starting point to better meet the dynamic market demand. (2) In this paper, the Elman neural network model is used to diagnose the probability of equipment failure and predict the uncertain market demand and build a multilayer game robust optimization model to solve the multiple uncertain problems such as the two-way uncertainty of production capacity and market demand and the uncertainty of matching relationship. (3) We integrate the multiobjective genetic algorithm on the basis of multiobjective particle swarm, which solves the problem that the traditional swarm intelligence algorithm is easy to fall into the local optimal solution. (4) Compared with the traditional equipment matching model, we consider the dynamic equipment matching efficiency maximization under multiple uncertain environments and combine the concept of shared manufacturing to innovatively give the idea of establishing dynamic configurable equipment, which reduces the waste of equipment capacity to a greater extent. (5) By building a multilayer supply and demand matching network of the system, the precise connection between supply and demand can be realized. Any changes in customer requirements and design changes can be quickly propagated and responded to in a timely manner in the entire system network, ultimately realizing the optimal dynamic equipment scheduling scheme and production scheme under the dynamic market demand.

The text is structured as follows: Section 2 is the problem description.  Section 3 is the diagnostic model based on the neural network system, which includes diagnosis of dynamic market demand based on the gray prediction neural network model (GM(1, 1)-Elman neural network), diagnosis of production capacity based on the neural network, analysis of model predictive control in the shared manufacturing model, and analysis of maximum matching effectiveness model.  Section 4 is a multilayer game analysis, which includes two-way uncertainty environment analysis, impact of uncertain demand on profit, the MPC model for dynamic equipment scheduling, and the maximum matching effectiveness model.  Section 5 is the multiobjective collaborative optimization model.  Section 6 is the introduction of the improved swarm intelligence algorithm (NSGA-II-MOPSO) and the performance test of the algorithm, as well as the introduction of the system flow based on neural network system diagnosis and matching performance in the shared manufacturing mode.  Section 7 is the model applications.  Section 8 summarizes the whole text.

## 2. Problem Description

**Figure 1:**
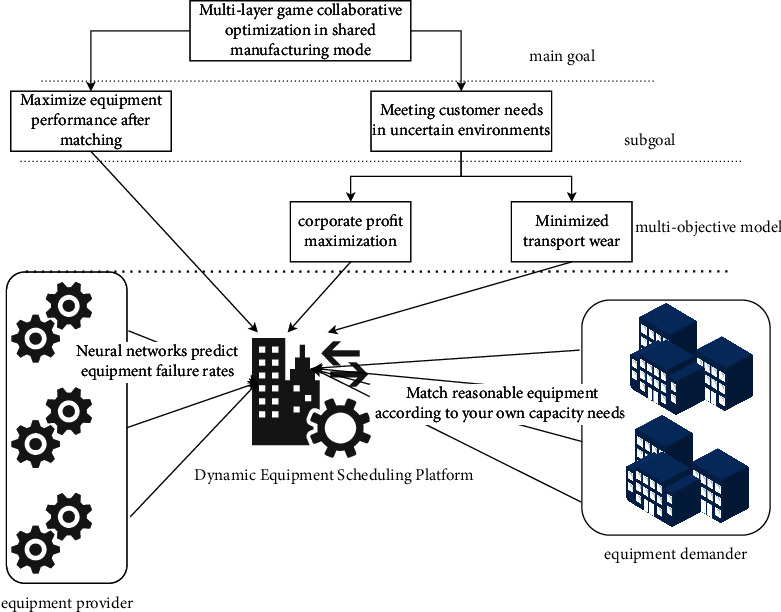
Optimization principle of the collaborative scheduling system considering dynamic equipment matching efficiency in shared manufacturing mode.

The self-profit of manufacturing enterprises under the sharing mode is related to the dynamic matching of idle resources. The production capacity of each manufacturing enterprise will vary with the implementation of different dynamic equipment scheduling schemes. Under the shared manufacturing mode, different from the uncertainty of market orders, the uncertainty of production capacity within the enterprise can be subdivided into two situations: uncertainty of equipment failure and uncertainty of matching efficiency. Therefore, the manufacturing capacity of manufacturing enterprises is constantly changing, and the uncertainty of internal production capacity of manufacturing enterprises and the uncertainty of demand in the market together constitute a two-way uncertainty environment. This two-way uncertainty environment will have a huge impact on the reasonable matching of dynamic resources in the manufacturing system, the profits of manufacturing enterprises, and the design of production planning schemes. In addition, in actual production, manufacturing enterprises can stimulate different degrees of manufacturing capacity through different matching schemes of idle equipment. At the same time, the matching efficiency of dynamic equipment in the production process should also be considered, so as to avoid unnecessary waste of labor and other costs, equipment damage, and other problems. In the shared manufacturing mode, the optimization principle of the dynamic equipment collaborative scheduling system is shown in Figure 1.

**Figure 2:**
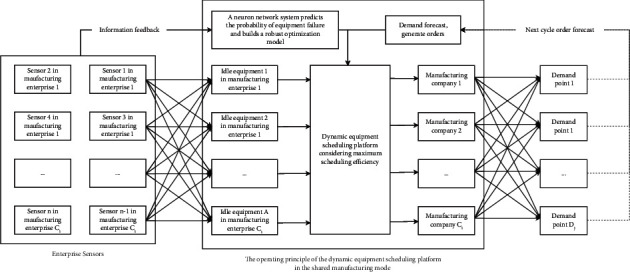
Cooperative scheduling system analysis model.

Aiming at the market uncertain demand problem mentioned above, this paper will study the establishment of demand under an uncertain demand environment, the efficiency of dynamic equipment matching, and the optimization of the collaborative scheduling system in manufacturing enterprises. The node types in this paper are enterprise, manufacturer, and wholesaler. Suppose the area contains *J* wholesalers. In order to better meet the large-scale personalized needs of customers, an enterprise establishes a dynamic equipment scheduling platform within the enterprise, which records the existing equipment information in the enterprise, the equipment load in the production process, and other equipment information at all times. At present, there are *I* subsidiaries in a manufacturing enterprise *M*_*t*_, and the quantity that can be used for equipment dispatching among the subsidiaries in the manufacturing enterprise is *A*. For the convenience of description, the following definitions are now made: the nodes in the network are represented by *T*、*I*、*K*、*J*、*A* an d *P*, respectively; among them, {1,2,…, *T*} ∈ *T* is the set of *M*_*t*_ nodes of the manufacturing enterprise, and {1,2,…, *I*} ∈ *I* is the set of *C*_*i*_ nodes of subsidiaries in the manufacturing enterprise. {1,2,…, *K*} ∈ *K* is the set of *W*_*k*_ nodes in the warehouse. {1,2,…, *J*} ∈ *J* is the set of *D*_*J*_ nodes of the wholesaler. {1,2,…, *P*} ∈ *P* is the set of *R*_*P*_ nodes of the demand point. The collaborative scheduling system analysis model is shown in Figure 2.

*C*_*ij*_^*tmα*^, *C*_*ij*_^*tmβ*^, an d *C*_*ij*_^*tmλ*^ represent the labor costs, material costs, and other costs corresponding to the production unit product *m* of the subsidiary *C*_*i*_ in the manufacturing enterprise *M*_*t*_, respectively. *V*_*ij*_^*tm*^ represents the unit sales price corresponding to the product *m* produced by the subsidiary *C*_*i*_ in the manufacturing enterprise *M*_*t*_. *V*_*k*_^*tm*^ represents the recycling price of the recycled product *m* in the warehouse *W*_*k*_ in the manufacturing enterprise *M*_*t*_. *V*_*j*_^*tm*^ represents the selling price of the remaining products of product *m* sold by wholesaler *D*_*j*_ in manufacturing enterprise *M*_*t*_ through other channels. *RP*_*jp*_^*tm*^ the retail price of product *m* by wholesaler *D*_*j*_ in manufacturer *M*_*t*_. *C*_*ij* *d*_^*tm*^ represents the unit product transportation unit price for the subsidiary *C*_*i*_ in the manufacturing enterprise *M*_*t*_ to choose the transportation method *d* to transport the product to the wholesaler *D*_*j*_, among them: *D* represents the set of transportation methods, *d* ∈ *D*. The actual arrival of goods received by the corresponding wholesaler *D*_*j*_ from subsidiary *C*_*i*_ is *Q*_*ijn*_^*tm*^=*Q*_*ij*_^*tm*^*∗*(1 − *e*_*ijd*_^*tm*^). The order quantity of the wholesaler is *Q*_*ji*_^*tm*^. *Q*_*pj*_^*tm*^ denotes the forecasted demand of wholesaler *D*_*j*_ for the corresponding demand point *R*_*P*_ with respect to product *m*. *Q*_*kmax*_^*tm*^ denotes the maximum storage capacity of the warehouse *W*_*k*_ with respect to product *m*. *Q*_*kn*_^*tm*^ denotes the existing storage capacity of the warehouse *W*_*k*_ with respect to product *m* and has *Q*_*kn*_^*tm*^ ∈ [0, *Q*_*kmax*_^*tm*^]. *Q*_*jkn*_^*tm*^ denotes the actual cargo arrival of wholesaler *D*_*j*_ regarding product *m* to the warehouse *W*_*k*_, and has *Q*_*jkn*_^*tm*^=*Q*_*jk*_^*tm*^(1 − *e*_*jkd*_^*tm*^), among them: *Q*_*jk*_^*tm*^, *e*_*jkd*_^*tm*^ denote the corresponding cargo volume and path wear rate, respectively. *Q*_*ki*_^*tm*^ denotes the warehouse *W*_*k*_ returns the remaining *Q*_*ki*_^*tm*^ indicates the amount of goods returned by the warehouse *W*_*k*_ to subsidiary *C*_*i*_ within manufacturing enterprise *M*_*t*_ for reprocessing. *Q*_*ijr*_^*tm*^, *Q*_*ijf*_^*tm*^ indicate the amount of goods reprocessed by subsidiary *C*_*i*_ within manufacturing enterprise *M*_*t*_ about product *m* and the amount of goods processed by the production line, respectively. *T*_*ijr*_^*tm*^ and *T*_*ijf*_^*tm*^ denote the time required to reprocess a unit of product at subsidiary *C*_*i*_ within manufacturing firm *M*_*t*_ regarding product *m* and the time required to process it through the production line, respectively. *Q*_*j*_^*m*^ denotes the volume of goods handled by wholesaler *D*_*j*_ regarding product *m* by other channels. *T*_*aii*′_^*tm*^ denotes the time required to move equipment from subsidiary *C*_*i*_ to subsidiary *C*_*i*′_ the sum of the transportation and adjustment times required. The total amount of products received by wholesaler *D*_*j*_, *Q*_*ijn*_^*tm*^, can be handled in three ways: (1) Wholesaler *D*_*j*_ sells the products to demand point *R*_*P*_ at *RP*_*jp*_^*tm*^, and the quantity is recorded as *Q*_*jp*_^*tm*^. (2) Wholesaler *D*_*j*_ returns the unsold products in (1) to warehouse *W*_*k*_, and the quantity is recorded as *Q*_*jk*_^*tm*^. (3) If there is still a surplus of products then wholesaler *D*_*j*_ sells them at a low price of *V*_*j*_^*tm*^, and the quantity is recorded as *Q*_*j*_.

## 3. Neural Network System Diagnosis Model

In this section, we propose a control strategy based on Elman Neural Network-Model Predictive Control (NNMPC). To enhance the supply-demand matching ability of subsidiaries to dynamic market demand and the enterprise's own interests, we design controllers with robust quality regarding equipment failure, equipment matching relationship, and dynamic market demand based on the shared manufacturing model.

### 3.1. System Diagnosis

#### 3.1.1. GM(1, 1)-Elman Neural Network Demand Diagnosis

**Figure 3:**
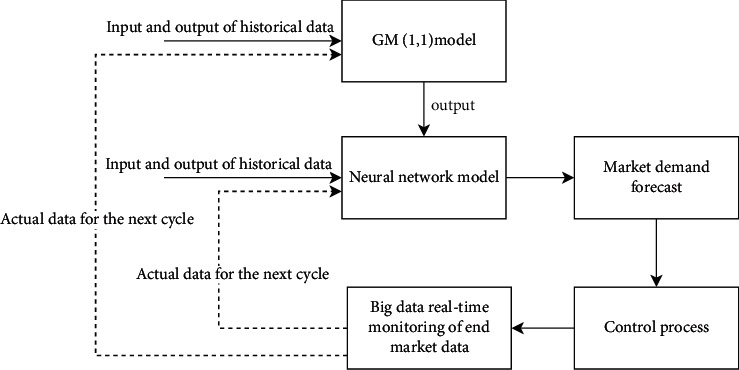
Dynamic prediction of the Elman neural network based on gray prediction.

Combined with the market environment, we conducted a big data analysis of the terminal of the market to know that the main factors that can be used to reflect the dynamic market demand are the following categories of factors: customer purchasing power, product market share, market terminal consumption records, and customer purchasing frequency and predict the next cycle of these four categories of factors through a gray prediction model (GM(1, 1)). We simulate the mapping relationship between each factor and market demand using a neural network model in combination with previous data, and then map the market demand in the next cycle from the predicted value of each factor in the next cycle. The specific process is shown in Figure 3; among them, the solid line indicates the market demand forecasting process in the current cycle and the dashed line indicates the data processing process in preparation for the next cycle.

We continuously train the neural network by updating the actual forecasting model through the process of monitoring the data at the terminal of the market. Thus, the terminal-of-market data will be updated twice in the process of dynamically forecasting market demand: once for the historical data in the gray forecasting model and once for the historical data in the neural network model. Among them, the specific implementation of gray forecasting is as follows:

Step 1: First, all gray sequences can be weakened by some kind of generation to show their randomness and regularity. We use the cumulative generation, let the initial data of each factor be *x*^(0)^=(*x*^(0)^(1), *x*^(0)^(2),……, *x*^(0)^(*n*)), the accumulation equation is *x*^(1)^(*n*)=*x*^0^(1)+*x*^0^(2)+……+*x*^0^(*n*), and then there is the cumulative data as *x*^(1)^=(*x*^1^(1), *x*^1^(2),……, *x*^1^(*n*)). The pro-valued generating number is obtained using *z*^1^(*n*)=*ax*^0^(*n*)+(1 − *a*)*x*^0^(*n* − 1), and *a* is called the generating coefficient. In particular, when *a*=0.5, the number is said to be the mean generating number.
Step 2: Second, the data are examined. Firstly, the rank ratio of the series is calculated, *λ*_*k*_=*x*^0^(*k* − 1)/*x*^0^(*k*), *k*=2,3,…, *n*, if all the rank ratios fall within the tolerable coverage interval *X*=(*e*^−2/*n*+1^, *e*^2/*n*+1^), then the series *x*^(0)^ can be modeled as GM(1, 1) for gray prediction. Otherwise, the data need to be transformed appropriately.
Step 3: Build the gray prediction model: define the gray derivative of *x*^(1)^ as *d*(*k*)=*x*^0^(*k*)=*x*^1^(*k*) − *x*^1^(*k* − 1), and let *z*^1^(*k*) be the neighbor-valued generating series of series *x*^1^(*k*), that is, *z*^1^(*k*)=*ax*^1^(*k*)+(1 − *a*)*x*^1^(*k* − 1). Thus, the model of the gray differential equation defining GM(1, 1) is *d*(*k*)+*az*^1^(*k*)=*b*, among them, *a* is called the development coefficient, *z*^1^(*k*) is called the whitening background value, and *b* is called the amount of gray action. We can obtain the set of equations as follows:
  (1) $$\begin{array}{c} \left\{\begin{array}{c} x^{0}\left(2\right)+ax^{1}\left(2\right)=b,\\ x^{0}\left(3\right)+ax^{1}\left(3\right)=b,\\ \ldots \\ x^{0}\left(n\right)+ax^{1}\left(n\right)=b. \end{array}\right. \end{array}$$
Listed according to the matrix method,
  (2) $$\begin{array}{c} u=\left[\begin{array}{c} a\\ b \end{array}\right],Y=\left[\begin{array}{c} x^{0}\left(2\right)\\ x^{0}\left(3\right)\\ \ldots \\ x^{0}\left(n\right) \end{array}\right],B=\left[\begin{array}{cc} -z^{1}\left(2\right) & 1\\ -z^{1}\left(3\right) & 1\\ \ldots & \ldots \\ -z^{1}\left(n\right) & 1 \end{array}\right]. \end{array}$$
Then, GM(1, 1) can be represented by *Y*=*Bu*. Eventually, we obtain the predicted value of
  (3) $$\begin{array}{c} x^{1}\left(t+1\right)=\left(x^{0}\left(1\right)-\frac{b}{a}\right)e^{-a}+\frac{b}{a},k=1,2,3,\ldots ,n-1. \end{array}$$
Step 4: Test the accuracy prediction of the gray model; we choose the posterior difference test method to test the data. The predicted ${\hat{x}}^{1}$ is obtained using cumulative subtraction to get ${\hat{x}}^{0}$, ${\hat{x}}^{0}\left(k\right)={\hat{x}}^{1}\left(k\right)-{\hat{x}}^{1}\left(k-1\right),k=2,3,\ldots ,n$. And calculate the residuals, $e\left(k\right)=x^{0}\left(k\right)-{\hat{x}}^{0}\left(k\right),k=1,2,\ldots ,n$. Calculate the variance *S*_1_ of the original sequence *x*^0^ and the variance *S*_2_ of the residual, and $S_{1}=1/n{\sum_{k=1}^{n}\left(x^{0}\left(k\right)-\overset{-}{x}\right)}^{2}$, $S_{2}=1/n{\sum_{k=1}^{n}\left(e\left(k\right)-\overset{-}{e}\right)}^{2}$. Calculate the posttest ratio, *C*=*S*_2_/*S*_1_ and check the observation. For specific values, please refer to Table 1.

We normalize the historical data of four factors, including customer purchasing power, product market share, terminal-of-market consumption records, customer purchasing frequency, and historical market demand data as the input and output values of the neural network model and train the neural network. Finally, the mapping relationship between each factor and the demand is obtained.

**Figure 4:**
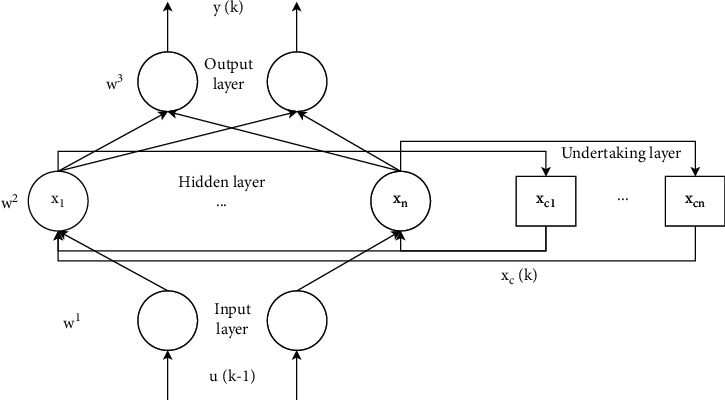
Elman neural network structure model.

Some studies have shown that, compared with the BP neural network in fault diagnosis, the Elman neural network has the characteristics of simple adjustment of structural parameters, short training time, and stable performance [48–51], and it is easier to find faults and diagnose them. The Elman neural network is a typical feedback neural network model that is widely used. It can generally be divided into four layers: input layer, hidden layer, successor layer, and output layer. The connection of its input layer, hidden layer, and output layer is similar to a feedforward network. The units of the input layer only play the role of signal transmission, and the units of the output layer play the role of weighting. The hidden layer unit has two types of excitation functions, linear and nonlinear, and the excitation function usually takes the sigmoid nonlinear function. The successor layer is used to memorize the output value of the hidden layer unit at the previous moment, which can be regarded as a delay operator with one-step delay. The output of the hidden layer is self-connected to the input of the hidden layer through the delay and storage of the successor layer. This self-connection method makes it sensitive to historical data. The addition of the internal feedback network increases the ability of the network itself to process dynamic information, so as to achieve the purpose of dynamic modeling. The Elman neural network structure is shown in Figure 4.

Practice has proved that Elman neural network has the following advantages:

1. It is sensitive to historical state, increases the ability to process state information, has adaptable time-varying characteristics, and can be dynamically modeled
2. It can approximate any nonlinear mapping with arbitrary precision, and it is not necessary to consider the specific form of the influence of external noise on the system
3. Effectiveness, widely used in practical problems
4. The associative memory function of the Elman neural network is better and more stable

The Elman neural network works as follows:

(a) Input layer and receiver layer
It is known that *x*_*it*_(*i*=1,2,…, *n*) is the input variable of the input layer node at time *t*, and *u*_*jt*_(*j*=1,2,…, *m*) is the node of the successor layer at time *t*. The corresponding expression is
  (4) $$\begin{array}{c} u_{jt}\left(k\right)=Z_{jt}\left(k-1\right),i=1,2,\ldots ,n,j=1,2,\ldots ,m, \end{array}$$
  (5) $$\begin{array}{c} net_{jt}\left(k\right)=\overset{n}{\underset{i=1}{\sum }}\omega_{ij}x_{it}\left(k-1\right)+\overset{m}{\underset{j=1}{\sum }}c_{j}u_{jt}\left(k\right). \end{array}$$
Among them, *ω*_*ij*_ is the weight value connecting the *i*-th input neuron node and the *j*-th hidden layer neuron node, and *Z*_*jt*_ is the neuron of the hidden layer at time *t*.
(b) Hidden layer
The relevant equation of the neurons in the hidden layer *Z*_*jt*_ at time *t* is expressed as follows:
  (6) $$\begin{array}{c} Z_{jt}\left(k\right)=f_{n}\left(\overset{n}{\underset{i=1}{\sum }}\omega_{ij}x_{it}\left(k\right)+\overset{m}{\underset{j=1}{\sum }}c_{j}u_{jt}\left(k\right)\right). \end{array}$$
The Sigmoid function expression of the hidden layer is *f*_*H*_(*x*)=1/(1+*e*^−*x*^), and *c*_*j*_ represents the weight value connecting the *j*-th node of the hidden layer to the node of the successor layer.
(c) Output layer
The output layer *y*_*t*+1_ can be expressed by the following equation:
  (7) $$\begin{array}{c} y_{t+1}\left(k\right)=f_{T}\left(\overset{m}{\underset{j=1}{\sum }}\nu_{j}Z_{ji}\left(k\right)\right). \end{array}$$
In the above equation, *ν*_*j*_ is the weight value connecting the *j*-th neuron of the hidden layer neuron to the output layer neuron.

**Figure 5:**
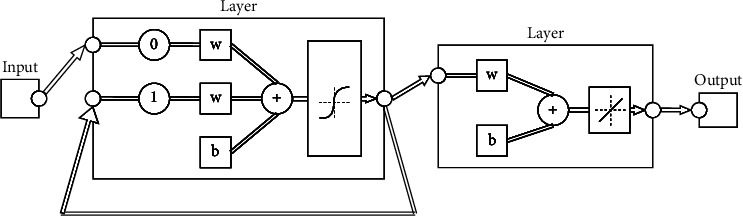
MATLAB structure diagram.

We predict data according to the basic structure of the Elman neural network system combined with MATLAB. The specific structure is shown in Figure 5.

#### 3.1.2. Elman Neural Network Productivity Diagnosis

**Figure 6:**
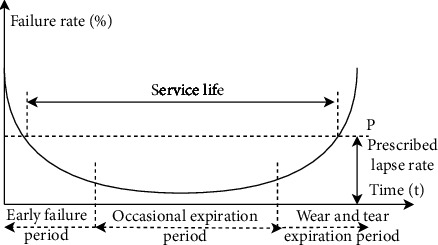
Bathtub curve.

Based on data collected from 500 enterprises, we know that the failure of most equipment is a function of time, and the typical failure curve is called the bathtub curve (failure rate curve). The bathtub curve shows that the reliability of the equipment changes with a certain law during the entire life cycle from investment to scrapping. We take the use time as the abscissa and the failure rate as a curve on the ordinate. The shape of the curve is high at both ends and low in the middle as shown in Figure 6.

**Figure 7:**
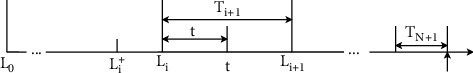
Change in service age of equipment over its life cycle.

The equipment fault establishment process combined with the neural network system is as follows:

Step 1: Calculate the respective service age regression factors and failure rate increment factors in the historical data according to different equipment models and performances. After analysis, we establish that the main factors affecting the two factors are the degree of equipment damage, the equipment load in operation, the proficiency of workers in operating the equipment, and the number of historical maintenance. The four factors are normalized and the Elman neural network algorithm is used to train the relationship between the four factors and the two factors. In the calculation of the next cycle after the training is completed, the service age regression factor and the failure rate increment factor are calculated by inputting the values corresponding to the four factors in the neural network model.
Step 2: We combine the service age regression factor and the failure rate increment factor established by the Elman neural network to determine the failure rate function of the equipment. The equipment failure rate function before and after preventive maintenance is as follows:
  (8) $$\begin{array}{c} \lambda_{i+1}\left(t\right)=\theta_{i}*\lambda_{i}\left(t+\delta_{i}*T_{i}\right),t\in \left(0,T_{i+1}\right). \end{array}$$
Among them, *i* is the number of preventive maintenance, *i*=1,2,…, *N*; *T*_*i*_ is the interval for the *i*-th preventive maintenance of the equipment; *λ*_*i*_(*t*) is the failure rate distribution function of the equipment before the *i*-th preventive maintenance; *λ*_0_(*t*) is the initial failure rate distribution function of the equipment; *θ*_*i*_ is the failure rate increment factor, *θ*_*i*_ > 1, whose value can be determined according to the historical data of the equipment operation combined with the actual situation; and *δ*_*i*_ is the service age regression factor, 0 < *δ*_*i*_ < 1. The value of *δ*_*i*_ is as follows:
  (9) $$\begin{array}{c} \delta_{i}={\left(a*\frac{C_{pmi}}{C_{\mathrm{pr}}}\right)}^{\tau ii^{\sigma }}. \end{array}$$
In the equation, *a* is the adjustment coefficient of equipment maintenance cost; *τ* is the time adjustment coefficient; and *σ* is the adjustment coefficient of learning effect, *σ*=ln *ψ*/ ln 2, and *ψ* is the percentage of experience curve given by experts based on experience judgment or estimation.
Step 3: Determine the maintenance interval of the equipment. Equipment preventive maintenance intervals in this document are based on the reliability of equipment components. First, the reliability threshold *R* of the equipment needs to be preset according to the characteristics of the equipment. When the reliability of the equipment reaches *R*, the equipment should be treated by preventive maintenance. According to the reliability function of the equipment,
  (10) $$\begin{array}{c} R_{i}\left(t\right)=\exp\;\left(-\int_{0}^{T_{i}}\lambda_{i}\left(t\right)\right). \end{array}$$
When equipment reaches its reliability threshold, then
  (11) $$\begin{array}{c} \exp\;\left(-\int_{0}^{T_{1}}\lambda_{1}\left(t\right)\right)=\exp\;\left(-\int_{0}^{T_{2}}\lambda_{2}\left(t\right)\right)=\ldots =\exp\;\left(-\int_{0}^{T_{i}}\lambda_{i}\left(t\right)\right)=R. \end{array}$$
By combining equations (8) and (11), the maintenance interval *T*_*i*_ of this type of equipment can be obtained.
Step 4: Establish the effective service age of the equipment. After the equipment undergoes preventive maintenance, the service age of the equipment will be reduced, and the retirement of the service age is related to the number of preventive maintenance of the equipment and the effect of preventive maintenance. Example: the effective service age before and after the first preventive maintenance is as follows:
  (12) $$\begin{array}{c} \left\{\begin{array}{c} L_{1}^{-}=T_{1},\\ L_{1}^{+}=\left(1-\delta_{1}\right)T_{1}. \end{array}\right. \end{array}$$
The effective service age before and after the second preventive maintenance is as follows:
  (13) $$\begin{array}{c} \left\{\begin{array}{c} L_{2}^{-}=L_{1}^{+}+T_{2}=\left(1-\delta_{2}\right)T_{1}+T_{2},\\ L_{2}^{+}=\left(1-\delta_{2}\right)L_{2}^{-}=\left(1-\delta_{1}\right)\left(1-\delta_{2}\right)T_{1}+\left(1-\delta_{2}\right)T. \end{array}\right. \end{array}$$
By analogy, the effective service age before and after the *i*-th preventive maintenance of the equipment is as follows:
  (14) $$\begin{array}{c} \left\{\begin{array}{c} L_{i}^{-}=L_{i-1}^{+}+T_{i}=\overset{i-1}{\underset{l=1}{\Pi }}\left(1-\delta_{l}\right)T_{1}+\overset{i-1}{\underset{l=2}{\Pi }}\left(1-\delta_{l}\right)T_{2}+\ldots +\left(1-\delta_{i-1}\right)T_{i-1}+T_{i},\\ L_{i}^{+}=\left(1-\delta_{i}\right)L_{i}^{-}=\overset{i}{\underset{l=1}{\Pi }}\left(1-\delta_{l}\right)T_{1}+\overset{i}{\underset{l=2}{\Pi }}\left(1-\delta_{l}\right)T_{2}+\ldots +\left(1-\delta_{i-1}\right)\left(1-\delta_{i}\right)T_{i-1}+\left(1-\delta_{i}\right)T_{i}. \end{array}\right. \end{array}$$
As shown in Figure 7, it can be seen from the analysis that the effective service age of the equipment in the *i*-th preventive maintenance interval is as follows:
  (15) $$\begin{array}{c} L_{i}\left(t\right)=L_{i}^{+}+t=L_{i}^{+}+\left(t-\overset{i}{\underset{s=1}{\sum }}T_{s}\right)=t+\left[\overset{i}{\underset{l=1}{\Pi }}\left(1-\delta_{l}\right)-1\right]T_{1}+\left[\overset{i}{\underset{l=2}{\Pi }}\left(1-\delta_{2}\right)-1\right]T_{2}+\ldots +\left[\overset{i}{\underset{l=i-1}{\Pi }}\left(1-\delta_{l}\right)-1\right]T_{i-1}-\delta_{i}T_{i}. \end{array}$$
The number of unexpected failures of the equipment during the *i*-th preventive maintenance interval is as follows:
  (16) $$\begin{array}{c} n_{i}=\int_{L_{i-1}^{+}}^{L_{i}^{-}}\lambda_{i}\left(t\right)dt. \end{array}$$
The total time required for maintenance over the life of the equipment is as follows:
  (17) $$\begin{array}{c} T_{m}=\overset{N+1}{\underset{i=1}{\sum }}t_{m}n_{i}=t_{m}\overset{N+1}{\underset{i=1}{\sum }}\int_{L_{i-1}^{+}}^{L_{i}^{-}}\lambda_{i}\left(t\right)dt. \end{array}$$
Among them, *T*_*m*_ is the total time required for equipment maintenance, and *t*_*m*_ is the maintenance time of one maintenance.
Step 5: Determine the validity of the equipment
The effectiveness of the equipment refers to the probability that the equipment is in a usable state during its life cycle. The effectiveness of the equipment is as follows:
  (18) $$\begin{array}{c} A=\lim_{n⟶\infty }\frac{A\left(t\right)}{T}=\frac{T-T_{m}}{T}=\frac{\overset{N+1}{\underset{i=1}{\sum }}T_{i}-t_{m}\sum_{i=1}^{N+1}\int_{L_{i-1}^{+}}^{L_{i}^{-}}\lambda_{i}\left(t\right)dt}{\overset{N+1}{\underset{i=1}{\sum }}T_{i}}. \end{array}$$

Combined with the equipment failure matrix, the failure probability of the corresponding type of equipment in the shared manufacturing platform is predicted. Due to the large number of equipments, the platform can set thresholds to achieve premaintenance of equipments. Assume that the threshold value corresponding to the equipment of the product *m* model *j* of the subsidiary *C*_*i*_ in the manufacturing enterprise *M*_*t*_ is *η*_*ij*_^*tm*^, *t* ∈ *T*, *m* ∈ *M*, *i* ∈ *I*, *j* ∈ *J*, and the corresponding equipment validity is *A*_*ij*_^*tm*^, *t* ∈ *T*, *m* ∈ *M*, *i* ∈ *I*, *j* ∈ *J*. Then, a judgment equation can be established to decide whether to perform premaintenance processing on the (*k*+1)-th equipment of this model. The specific judgment equation is as follows:

(19) $$\begin{array}{c} output\;a=\left\{\begin{array}{c} 1,if\left(A_{ij\left(k+1\right)}^{tm}>\eta_{ij}^{tm}\right)\\ 0,if\left(A_{ij\left(k+1\right)}^{tm}\leq \eta_{ij}^{tm}\right) \end{array}\right.\;,t\in T,m\in M,i\in I,j\in J. \end{array}$$

When the output result is *a*=1, the equipment needs to be premaintained. When the output result is *a*=0, it is considered that the performance of the equipment is good, and there is no need to perform premaintenance processing on the equipment. However, combined with the analysis of the actual situation, we found that when *a*=1, even if the equipment is prerepaired, some equipment will cause irreparable loss of production capacity due to depreciation and other reasons. That is, it is difficult for some equipment to be repaired to the previous equipment capacity. As a result, the uncertainty of equipment maintenance occurs. The specific calculation equation of equipment performance after premaintenance is as follows:

(20) $$\begin{array}{c} CO_{ijk}^{tm\nu }=CO_{ijk}^{tm\mu }+\Omega_{ijk}^{tm}*\Delta CO_{ijk}^{tm}\;,i\in I,j\in J,k\in K,t\in T,m\in M. \end{array}$$

And we have *CO*_*ijk*_^*tm*^=*CO*_*ijk*_^*tmμ*^+Δ*CO*_*ijk*_^*tm*^, *i* ∈ *I*, *j* ∈ *J*, *k* ∈ *K*, *t* ∈ *T*, *m* ∈ *M*. Among them, *CO*_*ijk*_^*tmν*^, Ω_*ijk*_^*tm*^, and Δ*CO*_*ijk*_^*tm*^, respectively, represent the equipment production capacity, maintenance efficiency, and production capacity loss of the *k*-th model *j* of the machine and equipment of the production product *m* of the subsidiary *C*_*i*_ in the manufacturing enterprise *M*_*t*_ after premaintenance treatment, and Ω_*ijk*_^*tm*^ ∈ [0,1].

## 4. Multilayer Game Analysis

### 4.1. Uncertainty about the Impact of Demand on Profits

First, based on the characteristics of the shared manufacturing model and combined with the actual production process, the profit (*f*^*tm*^(*x*)) of the product *m* in the manufacturing enterprise *M*_*t*_ can be divided into four parts, namely, sales revenue (*f*_1_^*tm*^(*x*)), equipment scheduling costs (*f*_2_^*tm*^(*x*)), production costs (*f*_3_^*tm*^(*x*)), and product transportation costs (*f*_4_^*tm*^(*x*)). The specific equation is expressed as follows:

(21) $$\begin{array}{c} f^{tm}\left(x\right)=f_{1}^{tm}\left(x\right)-f_{2}^{tm}\left(x\right)-f_{3}^{tm}\left(x\right)-f_{4}^{tm}\left(x\right),t\in T,m\in M. \end{array}$$

Sales revenue (*f*_1_^*tm*^(*x*)): the sales revenue obtained by manufacturing enterprise *M*_*t*_ from producing product *m* is expressed as follows:

(22) $$\begin{array}{c} f_{1}^{tm}\left(x\right)=\overset{I}{\underset{i=1}{\sum }}\overset{J}{\underset{j=1}{\sum }}\left(Q_{ij}^{tm}\right.*\left.V_{ij}^{tm}\right),t\in T,m\in M. \end{array}$$

Equipment scheduling costs (*f*_2_^*tm*^(*x*)): in the process of producing product *m* by manufacturing enterprise *M*_*t*_, the dispatching cost of dynamic equipment between subsidiaries during the dispatching process, including: equipment transportation cost (*C*_*aii*′_^*tm*^) and equipment installation cost (*C*_*ai*′_^*tm*^). The specific equation is expressed as follows:

(23) $$\begin{array}{c} f_{2}^{tm}\left(x\right)=\overset{A}{\underset{a=1}{\sum }}\overset{I}{\underset{i=1}{\sum }}\overset{I}{\underset{i^{\prime }=1}{\sum }}\left(C_{aii^{\prime }}^{tm}+C_{ai^{\prime }}^{tm}\right),t\in T,m\in M. \end{array}$$

Production costs (*f*_3_^*tm*^(*x*)): in the process of producing product *m* by manufacturing enterprise *M*_*t*_, the production costs of each subsidiary are divided into labor costs, material costs, and other costs. The specific equation is expressed as follows:

(24) $$\begin{array}{c} f_{3}^{tm}\left(x\right)=\overset{I}{\underset{i=1}{\sum }}\overset{J}{\underset{j=1}{\sum }}\left[Q_{ij}^{tm}*\left(C_{ij}^{tm\alpha }+C_{ij}^{tm\beta }+C_{ij}^{tm\lambda }\right)\right],t\in T,m\in M. \end{array}$$

Product transportation costs (*f*_4_^*tm*^(*x*)): after the manufacturing enterprise *M*_*t*_ produces the product *m*, each subsidiary selects the transportation mode *d* and the transportation cost incurred in the process of transporting the product to each wholesaler. The specific equation is expressed as follows:

(25) $$\begin{array}{c} f_{4}^{tm}\left(x\right)=\overset{I}{\underset{i=1}{\sum }}\overset{J}{\underset{j=1}{\sum }}\left(Q_{ij}^{tm}*C_{ij\;d}^{tm}\right),t\in T,m\in M,d\in D. \end{array}$$

To sum up, we have the total profit of manufacturing firm *M*_*t*_ producing product *m*:

(26) $$\begin{array}{c} f^{tm}\left(x\right)=\overset{I}{\underset{i=1}{\sum }}\overset{J}{\underset{j=1}{\sum }}Q_{ij}^{tm}-\overset{A}{\underset{a=1}{\sum }}\overset{I}{\underset{i=1}{\sum }}\overset{I}{\underset{i^{\prime }=1}{\sum }}\left(C_{aii^{\prime }}^{tm}+C_{ai^{\prime }}^{tm}\right)\\ -\overset{I}{\underset{i=1}{\sum }}\overset{J}{\underset{j=1}{\sum }}\left[Q_{ij}^{tm}*\left(C_{ij}^{tm\alpha }+C_{ij}^{tm\beta }+C_{ij}^{tm\lambda }\right)\right]-\overset{I}{\underset{i=1}{\sum }}\overset{J}{\underset{j=1}{\sum }}\left(Q_{ij}^{tm}*C_{ij\;d}^{tm}\right),t\in T,m\in M,d\in D. \end{array}$$

Correspondingly, the total profit equation of manufacturing enterprise *M*_*t*_ is as follows:

(27) $$\begin{array}{c} f^{t}\left(x\right)=\overset{M}{\underset{m=1}{\sum }}\left\{\overset{I}{\underset{i=1}{\sum }}\overset{J}{\underset{j=1}{\sum }}\left(Q_{ij}^{tm}*V_{ij}^{tm}\right)-\overset{A}{\underset{a=1}{\sum }}\overset{I}{\underset{i=1}{\sum }}\overset{I}{\underset{i^{\prime }=1}{\sum }}\left(C_{aii^{\prime }}^{tm}+C_{ai^{\prime }}^{tm}\right)\;\right.\\ -\overset{I}{\underset{i=1}{\sum }}\overset{J}{\underset{j=1}{\sum }}\left[Q_{ij}^{tm}*\left(C_{ij}^{tm\alpha }+C_{ij}^{tm\beta }+C_{ij}^{tm\lambda }\right)\right]-\overset{I}{\underset{i=1}{\sum }}\overset{J}{\underset{j=1}{\sum }}\left.\left(Q_{ij}^{tm}*C_{ijd}^{tm}\right)\right\},t\in T,d\in D. \end{array}$$

Composed of the profits of the abovementioned manufacturing enterprises, we found that after equipment scheduling, the manufacturing capacity of the manufacturing enterprises has been improved; but in the actual process, the predicted value often deviates from the actual value, resulting in some losses.

In the manufacturing process, as the actual demand of the demand point changes, the income and strategies of the corresponding subsidiaries and wholesalers will also change accordingly. In addition, since there is profit model analysis in the closed-loop supply chain system in the constructed model, it is necessary to establish the values of *V*_*ij*_^*tm*^ and *RP*_*jp*_^*tm*^. The specific definitions are as follows.

**Definition 1 .**

Combined with data, record the corresponding ∑_*p*=1_^*P*^*Q*_*pj*_^*tm*^ of each wholesaler as (*d*_1_, *d*_2_, *d*_3_,…, *d*_*p*_), and the corresponding probability is (*p*(*d*_1_), *p*(*d*_2_), *p*(*d*_3_),…, *p*(*d*_*p*_)).

We calculate the corresponding *P*(*D*) based on past data statistics. For specific values, please refer to Table 2. Among them, the corresponding *P*(*D*) calculation equation when the demand is *d*_*p*_ is as follows:

(28) $$\begin{array}{c} P\left(D\right)=1-\overset{P}{\underset{p=1}{\sum }}P\left(d_{p}\right). \end{array}$$

Chen and Ma analyzed the marginal pricing strategy in the supply chain model and put forward the following inferences [52].

**Inference 1.**

Combined with the uncertain demand environment, the wholesaler *R*_*k*_ has a unit overstock loss *C*_*o*_ and a unit shortage loss (opportunity loss) *C*_*u*_, among them, *Co*=*V*_*k*_^*tm*^ − *V*_*j*_^*tm*^, *Cu*=*RP*_*jp*_ − *V*_*k*_^*tm*^. Then, there are

(29) $$\begin{array}{c} RP_{jp}^{tm}=\frac{V_{k}^{tm}-V_{j}^{tm}}{P\left(D^{*}\right)}+V_{j}^{tm},j\in J,k\in K,p\in P. \end{array}$$

Li established the wholesale price under the condition of maximum expected profit by constructing a supply chain game model. Combined with equation (29), the following inferences are drawn [53].

**Inference 2.**

Under the premise of following the principle of the manufacturer's maximum profit, the manufacturer's wholesale price *WOG*_*ik*_ is as follows:

(30) $$\begin{array}{c} V_{ij}^{tm}=\frac{V_{j}^{tm}-V_{k}^{tm}}{2P\left(D^{*}\right)}+\frac{V_{k}^{tm}+Cu}{2},i\in I,j\in J,k\in K,t\in T,m\in M. \end{array}$$

### 4.2. MPC Model for Dynamic Equipment Scheduling

Under the shared manufacturing mode, there is uncertainty about the matching relationship between equipment and subsidiaries in the operation of the dynamic equipment scheduling platform, and it is difficult for enterprises to select the most suitable equipment according to their own conditions to meet the market demand in an uncertain environment. Secondly, it is difficult to determine the effect of equipment premaintenance. In the actual production process, it may happen that even if the equipment is prerepaired, equipment failure still occurs. In view of the current dynamic market problems, we choose to adopt predictive control (MPC) to enhance the robustness of the system. MPC is based on a process model and introduces a feedforward effect, enabling the system to react in advance and make appropriate changes to counteract expected disturbances.

**Figure 8:**
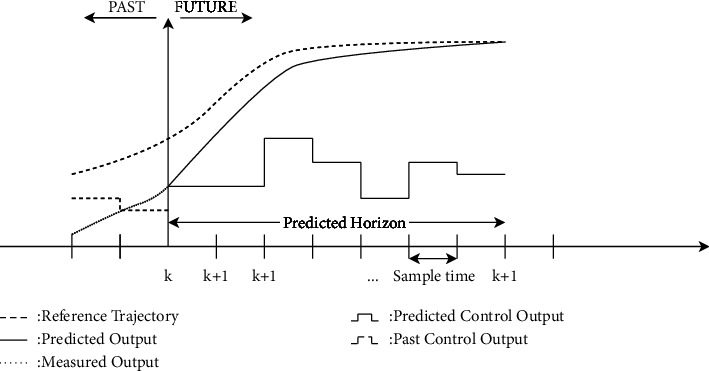
MPC algorithm implementation diagram.

The MPC algorithm mainly relies on two basic elements, an optimization tool and a system model. The former solves an optimal control problem to find control strategies that maximize the objective function, while the latter evaluates the future response of the system to these control strategies.  Figure 8 shows how the MPC algorithm works. The dashed line in Figure 8 is the reference trajectory, the dotted line is the actual evolution of the system, and the solid line is the evolution of the control action.

It can be seen from the above analysis that the production capacity of some equipment may change after premaintenance, and the maintenance effect of prerepaired equipment before the formal maintenance treatment is difficult to predict, so it is still prone to failure of some equipment in the production process. In addition, after some equipments are predicted, it is judged that no maintenance is required because *A*_*ij*(*k*+1)_^*tm*^ ≤ *η*_*ij*_^*tm*^, but there is still the possibility of failure in the production process, which will reduce the production capacity of the subsidiary. In order to solve the above problems, we propose to adopt a robust strategy for analysis, and carry out equipment scheduling with the goal of improving the subsidiary's production capacity in the worst case, namely, Ω_*ijk*_^*tm*^=0, *i* ∈ *I*, *j* ∈ *J*, *k* ∈ *K*, *t* ∈ *T*, *m* ∈ *M*. Let *CO*_*i*_^*tmv*^ and *CO*_*i*_^*tmv∗*^ represent the production capacity of product *m* produced by subsidiary *C*_*i*_ of manufacturing enterprise *M*_*t*_ after the equipment is repaired under normal and worst conditions, respectively. Then, there is the following equation:

(31) $$\begin{array}{c} \Delta_{i}^{tmv*}=CO_{i}^{tmv}-CO_{i}^{tmv*}. \end{array}$$

Among them, Δ_*i*_^*tmv∗*^ represents the corresponding loss of production capacity due to equipment failure.

**Figure 9:**
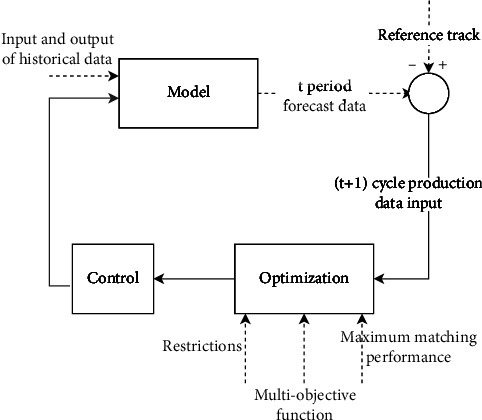
Predictive control optimization model based on neural network system diagnosis.

Manufacturing enterprises are often in dynamic changes. In order to ensure that subsidiaries can dynamically match changes in market demand, we build a predictive control optimization model as shown in Figure 9.

Each subsidiary predicts the actual demand of the corresponding demand point and transmits it to the resource agent after generating the information of each task agent. After receiving the information, the resource agent determines whether the idle equipment can be integrated with the scheduling agent and simulates the scheduling of the idle equipment by counting the number and model of the existing idle equipment, the manufacturing enterprises that can receive the equipment, the transportation route, and the process agent. After receiving the information, the scheduling agent establishes the capacity improvement range and cost change of each resource agent and generates the corresponding path plan to maximize the supply and demand matching ability of the system. In the production process of manufacturing enterprises, they can increase their own production capacity by receiving idle equipment, so as to better meet the dynamic market demand. The specific definitions are as follows.

**Definition 2 .**

There are a total of A sets of idle equipment that can be used for intersubsidiary scheduling; let *γ*^*m*^=(*γ*^1*m*^, *γ*^2*m*^,…, *γ*^*tm*^), *m* ∈ *M* means that each manufacturing enterprise produces product *m*. The manufacturing capacity of *γ*^*tm*^ represents the manufacturing capacity of the manufacturing enterprise *M*_*t*_ to produce product *m*. Δ*γ*_*aii*′_^*tm*^ represents the degree of increase in the manufacturing capacity of the manufacturing enterprise *M*_*t*_ after the equipment *a* is transferred from the subsidiary *C*_*i*_of the manufacturing enterprise *M*_*t*_ to the subsidiary *C*_*i*′_. Let *γ*^*mτ*^=(*γ*^1*mτ*^, *γ*^2*mτ*^,…, *γ*^*tmτ*^), *m* ∈ *M* represents the manufacturing capacity of each subsidiary *C*_*i*_ after each manufacturing enterprise receives idle equipment. *γ*^*tmτ*^ represents the manufacturing capacity of the manufacturing enterprise *M*_*t*_ to produce the product *m* after the manufacturing enterprise *M*_*t*_ receives the idle equipment. Among them,

(32) $$\begin{array}{c} \gamma^{tm\tau }=\gamma^{tm}+\overset{A}{\underset{a=1}{\sum }}\overset{I}{\underset{i=1}{\sum }}\overset{I}{\underset{i^{\prime }=1}{\sum }}\Delta \gamma_{aii^{\prime }}^{tm},t\in T,m\in M. \end{array}$$

After the dynamic equipment scheduling platform dispatches simulated idle equipment to each subsidiary, the increase in the production capacity of each subsidiary, the scheduling cost, and the multiobjective model are combined to establish the final scheduling plan and production plan. At the same time, a single subsidiary is allowed to receive multiple equipments, so it is defined as follows:

**Definition 3 .**

Let *CO*^*tm*^=(*CO*_1_^*tm*^, *CO*_2_^*tm*^,…, *CO*_*i*_^*tm*^), *t* ∈ *T*, *m* ∈ *M* represents the manufacturing capacity of each subsidiary in manufacturing enterprise *M*_*t*_ to produce product *m* before the scheduling occurs. *CO*_*i*_^*tm*^ represents the manufacturing capacity of the subsidiary *C*_*i*_ within the manufacturing enterprise *M*_*t*_ to produce product *m*. *CO*^*tmτ*^=(*CO*_1_^*tmτ*^, *CO*_2_^*tmτ*^,…, *CO*_*i*_^*tmτ*^), *t* ∈ *T*, *m* ∈ *M* represents the manufacturing capacity of each subsidiary in manufacturing enterprise *M*_*t*_ to produce product *m* after receiving idle equipment. *CO*_*i*_^*tmτ*^ represents the manufacturing capacity of the subsidiary *C*_*i*_ in the manufacturing enterprise *M*_*t*_ to produce the product *m* after receiving the idle equipment. Δ*CO*_*ai*_^*tm*^ represents the degree of increase in the manufacturing capacity of the subsidiary *C*_*i*_ after the subsidiary *C*_*i*_ in the manufacturing enterprise *M*_*t*_ receives the idle equipment *a*.

(33) $$\begin{array}{c} CO_{i}^{tm\tau }=CO_{i}^{tm}+\overset{A}{\underset{a=1}{\sum }}\Delta CO_{ai}^{tm},t\in T,m\in M. \end{array}$$

The platform establishes the equipment scheduling plan of each manufacturing enterprise according to the results of the simulated optimal plan, and allocates the task indicators reasonably. At the same time, after multiple subsidiaries in the manufacturing enterprise *M*_*t*_ receive the dispatch of multiple idle equipment, the overall production capacity of the manufacturing enterprise *M*_*t*_ will also increase. Hence the following inference:

**Inference 3.**

The manufacturing capacity of manufacturing enterprise *M*_*t*_ is *γ*^*tm*^=∑_*i*=1_^*I*^*CO*_*i*_^*tm*^, *t* ∈ *T*, *m* ∈ *M*, the production capacity of manufacturing enterprise *M*_*t*_ increases after receiving idle equipment. The degree is equal to the increase in the production capacity of each subsidiary in the manufacturing enterprise. The total production capacity change of the manufacturing enterprise *M*_*t*_ after receiving the idle equipment is as follows:

(34) $$\begin{array}{c} \gamma^{tm\tau }=\overset{I}{\underset{i=1}{\sum }}CO_{i}^{tm}+\overset{A}{\underset{a=1}{\sum }}\overset{I}{\underset{i=1}{\sum }}\Delta CO_{ai}^{tm},t\in T,m\in M. \end{array}$$

Combined with actual production, we can find that when the market suddenly fluctuates, the market demand forecasting model cannot respond in time and faces the problem of failure. If the production still follows the results of the forecasting model, it is very likely that the enterprise will suffer huge economic losses. The robust model makes up for this shortcoming. Before the market environment is fluctuated, the subsidiary can prepare for the fluctuation in advance according to the worst-case scenario of the market predicted for a long time. When the environment suddenly fluctuates, the subsidiary can respond in time to deal with this problem. Therefore, we construct a dual worst-case robust model to reduce the impact of uncertainty. The production quantity of each subsidiary is constantly changing, and the corresponding production plan, cargo distribution path, and distribution quantity are also changing at any time, so there are *Q*_*i*_^*tm*^=∑_*j*=1_^*J*^*Q*_*ij*_^*tm*^; thus, We take *Q*_*ij*_^*tm*^ as the system variable, and combine the robust model analysis to reversely establish the production quantity of each subsidiary to realize the adaptation of the market environment and the enterprise's manufacturing capacity. The corresponding supply and demand matching equation is as follows:

(35) $$\begin{array}{c} f_{1}\left(x\right)=\overset{A}{\underset{a=1}{\sum }}\overset{I}{\underset{i=1}{\sum }}\overset{I}{\underset{i^{\prime }=1}{\sum }}\left[CO_{i}^{tm}+\Delta CO_{aii^{\prime }}^{tm}\right]-\overset{J}{\underset{j=1}{\sum }}Q_{j,e}^{tm},t\in T,m\in M,e\in E. \end{array}$$

In addition, due to equipment failure, some equipment is still difficult to restore the original equipment production capacity after maintenance, so the corresponding supply and demand matching equation is changed to the following formula:

(36) $$\begin{array}{c} f_{1}\left(x\right)=\overset{A}{\underset{a=1}{\sum }}\overset{I}{\underset{i=1}{\sum }}\overset{I}{\underset{i^{\prime }=1}{\sum }}\left[CO_{i}^{tm}+\Delta CO_{aii^{\prime }}^{tm}\right]-\overset{I}{\underset{i=1}{\sum }}\overset{J}{\underset{j=1}{\sum }}\overset{K}{\underset{k=1}{\sum }}\Delta CO_{ijk}^{tm}-\overset{I}{\underset{i=1}{\sum }}\overset{J}{\underset{j=1}{\sum }}Q_{ij}^{tm},t\in T,m\in M. \end{array}$$

Combined with the analysis of existing market conditions, there is a robust model as follows:

(37) $$\begin{array}{c} \underset{i、i^{\prime }\in I,j\in J}{minmax}\left\{\left.\frac{f_{1}\left(Q_{ij}^{\alpha tm},\Delta CO_{aii^{\prime }}^{\alpha tm}\right)-f_{1}\left(Q_{ij}^{\alpha tm*},\Delta CO_{aii^{\prime }}^{\alpha tm}\right)}{f_{1}\left(Q_{ij}^{\alpha tm},\Delta CO_{aii^{\prime }}^{\alpha tm}\right)}\right|g\left(Q_{ij}^{\alpha tm*},CO_{i}^{\alpha tm}\right)\leq 0\right\},t\in T,m\in M,\forall \alpha . \end{array}$$

Among them, *Q*_*ij*_^*αtm∗*^ represents the delivery volume of product *m* delivered by enterprise *C*_*i*_ to *D*_*j*_ under the current uncertain environment. From the above analysis, when the market environment changes greatly and there are many possibilities, we require the manufacturing scheme to maintain strong robustness in various situations to reduce corporate losses. Therefore, we propose to adopt the objective function of equation (38) to improve the robustness of the system under complex environmental influences. If complex environmental factors affect the system and the subsidiary has a certain basis for environmental forecasting, we can convert equation (37) into equation (38) by combining multiple worst-case market demand equation as follows:

(38) $$\begin{array}{c} \min_{i,i^{\prime }\in I,j\in J}\left\{\left.\frac{f_{1}\left(Q_{ij}^{tm},\Delta CO_{aii^{\prime }}^{tm}\right)-f_{1}\left(Q_{ij}^{tm*},\Delta CO_{aii^{\prime }}^{tm}\right)}{f_{1}\left(Q_{ij}^{tm},\Delta CO_{aii^{\prime }}^{tm}\right)}\right|g\left(Q_{ij}^{tm*},CO_{i}^{tm}\right)\leq 0\right\},t\in T,m\in M. \end{array}$$

In the actual plan selection, the enterprise can select the specific plan in the Pareto surface and produce it according to the possibility of dynamic fluctuations in the current market environment. We combine the multiobjective model and the NSGA-II-MOPSO swarm intelligence algorithm (multiobjective genetic-particle swarm intelligence algorithm) to solve the optimal production plan. Thus, we can construct a feedback correction system to realize the system rolling optimization process within the model predictive control.

In actual production, we find that there are still production deviations due to machine failures and other reasons. In order to avoid its impact on production capacity, there are

(39) $$\begin{array}{c} \Lambda_{ie}^{tm\Gamma }=Q_{ie}^{tm*}-Q_{ie}^{tm\Gamma },i\in I,e\in E,t\in T,m\in M. \end{array}$$

Among them, Δ_*ie*_^*tm*Γ^ represents the actual production deviation value in stage *e*, *Q*_*ie*_^*tm∗*^, and *Q*_*ie*_^*tm*Γ^ represent the planned value and actual production value of the subsidiary's robust plan in stage *e*, respectively. If there is a production deviation in stage *e*, the deviation should be compensated in the next production process, there is an equation as follows:

(40) $$\begin{array}{c} Q_{i\left(e+1\right)}^{tm\gamma }=Q_{i\left(e+1\right)}^{tm*}+\Lambda_{ie}^{tm\Gamma }. \end{array}$$

Among them, *Q*_*i*(*e*+1)_^*tmγ*^ represents the planned production plan value of subsidiary *C*_*i*_ in the (*e* + 1) stage. Therefore, we found that in the actual production process, the internal rolling optimization method of the subsidiary can better solve the problem of output deviation between the production plan and the actual production.

### 4.3. Maximum Matching Performance Model

In an uncertain environment, it is difficult to achieve an absolute balance between supply and demand, and there is often an oversupply or undersupply after the idle equipment is dispatched. Therefore, in order to accurately grasp the idle equipment scheduling and resource usage and combine the characteristics of production planning and multisource and multiequipment scheduling, we now analyze the supply and demand relationship before and after the idle equipment scheduling. Δ^*tm*^ represents the maximum supply and demand matching efficiency function of idle equipment. We propose the following theorem:

**Theorem 4 .**

Idle equipment dispatch occurs before the oversupply, so there is ∑_*j*=1_^*J*^∑_*i*=1_^*I*^*Q*_*ji*_^*tm*^ < *γ*^*tm*^, *t* ∈ *T*, *m* ∈ *M*; then there is no need for equipment dispatch; at this time, the manufacturing enterprise has the capacity to meet the corresponding demand, and the supply and demand matching capacity can reach the maximum. Therefore, the current manufacturing enterprise capacity is *γ*^*tmτ*^=*γ*^*tm*^=∑_*i*=1_^*I*^*CO*_*i*_^*tm*^ and ∑_*a*=1_^*A*^∑_*i*=1_^*I*^Δ*CO*_*ai*_^*tm*^=0, *t* ∈ *T*, *m* ∈ *M*. At this time, the waste of production materials should be considered and should be achieved to meet the wholesaler's order quantity while minimizing the waste of capacity, so there is an objective function as follows:

(41) $$\begin{array}{c} \min\;\Delta^{tm}=\min\;\left\{\left|\gamma^{tm\tau }-\overset{J}{\underset{j=1}{\sum }}\overset{I}{\underset{i=1}{\sum }}Q_{ji}^{tm}\right|\right\}=\min\;\left\{\left|\overset{I}{\underset{i=1}{\sum }}CO_{i}^{tm}-\overset{J}{\underset{j=1}{\sum }}\overset{I}{\underset{i=1}{\sum }}Q_{ji}^{tm}\right|\right\},t\in T,m\in M. \end{array}$$

**Theorem 5 .**

Supply exceeds demand before idle equipment scheduling occurs, and oversupply after idle equipment scheduling occurs. Before the scheduling occurs because the demand-supply matching does not achieve equilibrium, that is, ∑_*j*=1_^*J*^∑_*i*=1_^*I*^*Q*_*ji*_^*tm*^ < *γ*^*tm*^, *t* ∈ *T*, *m* ∈ *M*, so the idle equipment scheduling should occur, and the demand-oriented scheduling of idle equipment occurs in the demand-supply matching model, so the idle equipment scheduling should occur at this time. Then we have *γ*^*tmτ*^=∑_*i*=1_^*I*^*CO*_*i*_^*tmτ*^=[∑_*i*=1_^*I*^*CO*_*i*_^*tm*^+∑_*a*=1_^*A*^∑_*i*=1_^*I*^Δ*CO*_*ai*_^*tm*^]. However, after the occurrence of idle equipment scheduling there are ∑_*j*=1_^*J*^∑_*i*=1_^*I*^*Q*_*ji*_^*tm*^ < *γ*^*tmτ*^. At this time, the waste of production materials should be considered, so there is an objective function.

(42) $$\begin{array}{c} \min\;\Delta^{tm}=\min\;\left\{\left|\gamma^{tm\tau }-\overset{J}{\underset{j=1}{\sum }}\overset{I}{\underset{i=1}{\sum }}Q_{ji}^{tm}\right|\right\}=\min\;\left\{\left|\left[\overset{I}{\underset{i=1}{\sum }}CO_{i}^{tm}+\overset{A}{\underset{a=1}{\sum }}\overset{I}{\underset{i=1}{\sum }}\Delta CO_{ai}^{tm}\right]-\overset{J}{\underset{j=1}{\sum }}\overset{I}{\underset{i=1}{\sum }}Q_{ji}^{tm}\right|\right\},t\in T,m\in M. \end{array}$$

**Theorem 6 .**

Idle equipment dispatch occurs before the supply exceeds demand ∑_*j*=1_^*J*^∑_*i*=1_^*I*^*Q*_*ji*_^*tm*^ > *γ*^*tm*^, *t* ∈ *T*, *m* ∈ *M*, as stated in Theorem 5, so we have *γ*^*tmτ*^=∑_*i*=1_^*I*^*CO*_*i*_^*tmτ*^=[∑_*i*=1_^*I*^*CO*_*i*_^*tm*^+∑_*a*=1_^*A*^∑_*i*=1_^*I*^Δ*CO*_*ai*_^*tm*^]. After the dispatch of idle equipment, there is still an oversupply of ∑_*j*=1_^*J*^∑_*i*=1_^*I*^*Q*_*ji*_^*tm*^ > *γ*^*tmτ*^, and the maximum capacity should be considered to solve the wholesaler demand problem to the maximum extent, and the objective function should be as follows:

(43) $$\begin{array}{c} \min\;\Delta^{tm}=\min\left\{\left|\overset{J}{\underset{j=1}{\sum }}\overset{I}{\underset{i=1}{\sum }}Q_{ji}^{tm}-\gamma^{tm\tau }\right|\right\}=\min\;\left\{\left|\overset{J}{\underset{j=1}{\sum }}\overset{I}{\underset{i=1}{\sum }}Q_{ji}^{tm}-\left[\overset{I}{\underset{i=1}{\sum }}CO_{i}^{tm}+\overset{A}{\underset{a=1}{\sum }}\overset{I}{\underset{i=1}{\sum }}\Delta CO_{ai}^{tm}\right]\right|\right\},t\in T,m\in M. \end{array}$$

In summary, then there are

(44) $$\begin{array}{c} \min\left|\Delta^{tm}\right|=\left\{\begin{array}{c} \min\;\left\{\left|\gamma^{tm\tau }-\overset{J}{\underset{j=1}{\sum }}\overset{I}{\underset{i=1}{\sum }}Q_{ji}^{tm}\right|\right\}=\min\;\left\{\left|\overset{I}{\underset{i=1}{\sum }}CO_{i}^{tm}-\overset{J}{\underset{j=1}{\sum }}\overset{I}{\underset{i=1}{\sum }}Q_{ji}^{tm}\right|\right\},if\overset{J}{\underset{j=1}{\sum }}\overset{I}{\underset{i=1}{\sum }}Q_{ji}^{tm}\leq \gamma^{tm}\\ \min\;\left\{\left|\overset{J}{\underset{j=1}{\sum }}\overset{I}{\underset{i=1}{\sum }}Q_{ji}^{tm}-\gamma^{tm\tau }\right|\right\}=\min\;\left\{\left|\overset{J}{\underset{j=1}{\sum }}\overset{I}{\underset{i=1}{\sum }}Q_{ji}^{tm}-\left[\overset{I}{\underset{i=1}{\sum }}CO_{i}^{tm}+\overset{A}{\underset{a=1}{\sum }}\overset{I}{\underset{i=1}{\sum }}\Delta CO_{ai}^{tm}\right]\right|\right\},if\overset{J}{\underset{j=1}{\sum }}\overset{I}{\underset{i=1}{\sum }}Q_{ji}^{tm}>\gamma^{tm}, \end{array}\right.\\ t\in T,m\in M. \end{array}$$

Among them, *γ*^*tmτ*^=∑_*i*=1_^*I*^*CO*_*i*_^*tm*^+∑_*a*=1_^*A*^∑_*i*=1_^*I*^Δ*CO*_*ai*_^*tm*^, *t* ∈ *T*, *m* ∈ *M*. In turn, the selection and scheduling of the corresponding equipment is realized.

## 5. Multiobjective Collaborative Optimization Model

After equipment scheduling occurs, the productivity improvement of each resource agent is simulated and counted. In this paper, a mathematical model is constructed to minimize maximum supply and demand deviations and maximize corporate profits as the multiobjective. The goal of maximum corporate profit is a key link to achieve the goal of corporate benefit under the shared manufacturing model. Combined with actual analysis, we also establish a robust optimization model to ensure that the subsidiary can still ensure the balance of supply and demand in the system in the worst case. The specific objective function is as follows:

(45) $$\begin{array}{c} \underset{i、i^{\prime }\in I,j\in J}{minmax}\left\{\left.\frac{f_{1}\left(Q_{ij}^{\alpha tm},\Delta CO_{aii^{\prime }}^{\alpha tm}\right)-f_{1}\left(Q_{ij}^{\alpha tm*},\Delta CO_{aii^{\prime }}^{\alpha tm}\right)}{f_{1}\left(Q_{ij}^{\alpha tm},\Delta CO_{aii^{\prime }}^{\alpha tm}\right)}\right|g\left(Q_{ij}^{\alpha tm*},CO_{i}^{\alpha tm}\right)\leq 0\right\},t\in T,m\in M,\forall \alpha , \end{array}$$

(46) $$\begin{array}{c} \max\;f^{tm}\left(x\right)=\overset{I}{\underset{i=1}{\sum }}\overset{J}{\underset{j=1}{\sum }}\left(Q_{ij}^{tm}*V_{ij}^{tm}\right)-\overset{A}{\underset{a=1}{\sum }}\overset{I}{\underset{i=1}{\sum }}\overset{I}{\underset{i^{\prime }=1}{\sum }}\left(C_{aii^{\prime }}^{tm}+C_{ai^{\prime }}^{tm}\right) \end{array}$$

Among them,

(47) $$\begin{array}{c} V_{ij}^{tm}=\frac{V_{j}^{tm}-V_{k}^{tm}}{2P\left(D^{*}\right)}+\frac{V_{k}^{tm}+Cu}{2},i\in I,j\in J,k\in K,t\in T,m\in M. \end{array}$$

The constraints are as follows:

(48) $$\begin{array}{c} \overset{A}{\underset{a=1}{\sum }}\overset{I}{\underset{i=1}{\sum }}\overset{I}{\underset{i^{\prime }=1}{\sum }}x_{aii^{\prime }}^{tm}\leq A, \end{array}$$

(49) $$\begin{array}{c} \lambda i+1\left(t\right)=\theta_{i}*\lambda_{i}\left(t+\delta_{i}*T_{i}\right),t\in \left(0,T_{i+1}\right), \end{array}$$

(50) $$\begin{array}{c} \delta_{i}={\left(a*\frac{C_{pmi}}{C_{\mathrm{pr}}}\right)}^{\tau ii^{\sigma }}, \end{array}$$

(51) $$\begin{array}{c} \left\{\begin{array}{c} L_{i}^{-}=L_{i-1}^{+}+T_{i}=\overset{i-1}{\underset{l=1}{\Pi }}\left(1-\delta_{l}\right)T_{1}+\overset{i-1}{\underset{l=2}{\Pi }}\left(1-\delta_{l}\right)T_{2}+\ldots +\left(1-\delta_{i-1}\right)T_{i-1}+T_{i},\\ L_{i}^{+}=\left(1-\delta_{i}\right)L_{i}^{-}=\overset{i}{\underset{l=1}{\Pi }}\left(1-\delta_{l}\right)T_{1}+\overset{i}{\underset{l=2}{\Pi }}\left(1-\delta_{l}\right)T_{2}+\ldots +\left(1-\delta_{i-1}\right)\left(1-\delta_{i}\right)T_{i-1}+\left(1-\delta_{i}\right)T_{i}, \end{array}\right. \end{array}$$

(52) $$\begin{array}{c} A=\lim_{n⟶\infty }\frac{A\left(t\right)}{T}=\frac{T-T_{m}}{T}=\frac{\overset{N+1}{\underset{i=1}{\sum }}T_{i}-t_{m}\overset{N+1}{\underset{i=1}{\sum }}\int_{L_{i-1}^{+}}^{L_{i}^{-}}\lambda_{i}\left(t\right)dt}{\overset{N+1}{\underset{i=1}{\sum }}T_{i}}, \end{array}$$

(53) $$\begin{array}{c} CO_{ijk}^{tm\nu }=CO_{ijk}^{tm\mu }+\Omega_{ijk}^{tm}*\Delta CO_{ijk}^{tm}\;,i\in I,j\in J,k\in K,t\in T,m\in M, \end{array}$$

(54) $$\begin{array}{c} Q_{ijn}^{tm}=\left(Q_{ijr}^{tm}+Q_{ijf}^{tm}\right)*\left(1-e_{ij\;d}^{tm}\right),i\in I,j\in J,d\in D,t\in T,m\in M, \end{array}$$

(55) $$\begin{array}{c} RP_{jp}^{tm}>V_{ij}^{tm}>V_{k}^{tm}>V_{j}^{tm},i\in I,j\in J,k\in K,p\in P,t\in T,m\in M, \end{array}$$

(56) $$\begin{array}{c} \overset{I}{\underset{i=1}{\sum }}\left(Q_{ijr}^{tm}*T_{ijr}^{tm}\right)+\overset{I}{\underset{i=1}{\sum }}\left(Q_{ijf}^{tm}*T_{ijf}^{tm}\right)+\overset{I}{\underset{i=1}{\sum }}\left(Q_{ij}^{tm}*T_{ijd}^{tm}\right)+\overset{A}{\underset{a=1}{\sum }}\overset{I}{\underset{i=1}{\sum }}\overset{I}{\underset{i^{\prime }=1}{\sum }}T_{aii^{\prime }}\leq T_{j\;\max}, \end{array}$$

(57) $$\begin{array}{c} Q_{jkn}^{tm}=Q_{jk}^{tm}\left(1-e_{kjd}^{tm}\right),j\in J,k\in K,t\in T,m\in M, \end{array}$$

(58) $$\begin{array}{c} Q_{ij}^{tm}=Q_{ijr}^{tm}+Q_{ijf}^{tm},i\in I,j\in J,t\in T,m\in M, \end{array}$$

(59) $$\begin{array}{c} \overset{I}{\underset{i=1}{\sum }}Q_{ijn}^{tm}=\overset{P}{\underset{p=1}{\sum }}Q_{jp}^{tm}+\overset{K}{\underset{k=1}{\sum }}Q_{jk}^{tm}+Q_{j}^{m},j\in J,t\in T,m\in M, \end{array}$$

(60) $$\begin{array}{c} Q_{kn}^{tm}+\overset{J}{\underset{j=1}{\sum }}Q_{jkn}^{tm}\leq Q_{k\;\max}^{tm},k\in K,t\in T,m\in M, \end{array}$$

(61) $$\begin{array}{c} \overset{I}{\underset{i=1}{\sum }}Q_{ki}^{tm}\leq Q_{kn}^{tm},k=1,\ldots ,K, \end{array}$$

(62) $$\begin{array}{c} \overset{K}{\underset{k=1}{\sum }}Q_{ik}\leq CO_{i}^{tm}+\overset{A}{\underset{a=1}{\sum }}\Delta CO_{ai}^{tm},i\in I,t\in T,m\in M, \end{array}$$

(63) $$\begin{array}{c} x_{aii^{\prime }}^{tm}=\left\{\begin{array}{c} 1,i\;dl\;e\;equipment\;a\;is\;di\;spatche\;d\;from\;manufacturing\;enterprise\;C_{i}\;to\;C_{i^{\prime }},\\ 0, \end{array}\right. \end{array}$$

Equation (53) indicates that the total number of equipments received in the manufacturing enterprise *M*_*t*_ should not be greater than the total number of equipments in the platform. Equation (54) indicates that the goods actually received by wholesaler *D*_*j*_ should be equal to the amount of goods shipped by each subsidiary *C*_*i*_ minus the amount of wear-and-tear goods. Equation (55) indicates the relationship between the four prices. Equation (56) indicates that the sum of the processing time and the goods transportation time of the subsidiaries in each manufacturing enterprise should not be greater than the goods delivery time of the wholesaler *D*_*j*_. Equation (57) indicates that the actual amount of goods arriving at the warehouse should be equal to the amount of remaining goods returned by each wholesaler *D*_*j*_ minus the amount of wear and tear of goods in the journey. Equation (58) indicates that the total production amount of product *m* from the subsidiary *C*_*i*_ within the manufacturing enterprise *M*_*t*_ is equal to the sum of the production line processing volume and the reprocessed storage product volume. Equation (59) indicates that there are three ways for wholesaler *D*_*j*_ to deal with the actual quantity of goods arriving: selling to the corresponding customer, returning the product to the warehouse, and selling in other ways. Equation (60) indicates that the sum of the quantity of goods returned by each wholesaler *D*_*j*_ to warehouse *W*_*k*_ and the existing storage capacity of warehouse *W*_*k*_ should not be greater than the maximum storage capacity of warehouse *W*_*k*_ for product *m*. Equation (61) indicates that the total amount of goods transported by warehouse *W*_*k*_ to each subsidiary should not be greater than the existing stock of product *m* in warehouse *W*_*k*_. The equation (62) indicates that the total amount of goods delivered by the subsidiary *C*_*i*_ in the manufacturing enterprise *M*_*t*_ to each wholesaler *D*_*j*_ should not be greater than the current production capacity of the subsidiary *C*_*i*_ after equipment scheduling. Equation (63) indicates a 0-1 variable.

## 6. Improved Swarm Intelligence Algorithm

Assuming an *N*-dimensional space, the solution composition of the problem can be indicated by an *n* -dimensional vector. In each generation of the algorithm, the current best position found by particle *q* itself is *pop*(*i*).*Best*.*Position* and a randomly selected nondominated solution by the roulette operator is used as the global optimal solution *lea* *de* *r*.*Position*. If the vectors *pop*(*i*).*Position* and *pop*(*i*).*Velocity* are used to denote the particle *q*'s position and velocity, respectively, in the algorithm position and velocity, the following two equations describe the process of updating the particle velocity and position vectors:

(64) $$\begin{array}{c} \text{pop}\left(i\right).\text{Velocity}=\omega *\text{pop}\left(i\right).\text{Velocity}+c1*r\text{and}\left(\text{Varsize}\right).*\left(\text{pop}\left(i\right).\text{Best}.\text{Position}\right) \end{array}$$

(65) $$\begin{array}{c} \text{pop}\left(i\right).\text{Position}=\text{pop}\left(i\right).\text{Position}+\text{pop}\left(i\right).\text{Velocity}. \end{array}$$

Among them, *ω* is the inertia coefficient; *c*1 and *c*2 are the positive coefficients, which are acceleration factors and used to change the moving distance of particles when they follow the optimal direction of their own best position and the overall best position. Increase the convergence speed of the algorithm while jumping out of the local optimal limit. rand(VarSize) is an independent random number between [0, 1] that obeys a uniform distribution. The stopping criterion of the algorithm is the maximum number of iterations.

### 6.1. Particle Encoding

  Step 1: Set the algorithm parameters. Population number *n*pop, nondominated solution population number *n*rep, iteration times MaxIt, individual and group coefficients *c*1, *c*2, and the expanded network size *n*Grid of each objective function solution set domain.
  Step 2: Initialize the particle population, including the position, speed, and other information of the particle swarm to construct a particle population with a structure.
  Step 3: Calculate the particle objective function value, determine the individual optimal position *pop*(*i*).*Position* of the particle individual, and calculate the optimal fitness value pop(*i*).Best.Cost.
  Step 4: Generate the nondominated solution set and start the iteration.
  Step 5: Update the particle position pop(*i*).Position and particle velocity pop(*i*).Velocity in the particle swarm
  Step 6: When the particle satisfies the constraints, go to step 7, and if it does not meet the constraints, go to step 8.
  Step 7: Calculate the evaluation index of each particle and update the individual optimal particle using the dominance relationship, go to step 11.
  Step 8: Enter the *while* loop, convert the particle swarm particle pop(*i*) into the genetic particle chromo(*i*), and generate the parent population chromo_parent.
  Step 9: The strategy of selection, crossover, and mutation is adopted for the genetic particle chromo(*i*), and the progeny population chromo_off is obtained.
  Step 10: After merging the parent population chromo_parent with the child population chromo_off and calculating the nondominated ranking and crowding degree, the nondominated genetic particles are retained to generate a nonbranched set. Exit the loop until the *while* loop is satisfied, and convert the genetic particle chromo(*i*) into the particle swarm particle pop(*i*). Proceed to step 11.
  Step 11: Update the nondominated solution set, truncate the nondominated solution set, and retain *nrep* optimal solutions
  Step 12: Determine whether the corresponding number of iterations is satisfied. If not, loop to step (3). If so, end the iteration and draw the particles corresponding to the corresponding nondominated solution set to generate a pareto surface and output the nondominated solution set.

In addition, after generating *i∗j* particles *Q*_*ij*_ indicating the transportation volume of each path in each production range, the corresponding robustness of supply-demand matching and manufacturing firm profit are calculated. For the convenience of plotting, the manufacturing firm profits are recorded by taking negative values. If the value of the corresponding supply-demand matching capability and customer value evaluation function of the particle generated in the (*i*+1)−th iteration is less than the corresponding supply-demand matching capability and manufacturing enterprise profit of the particle generated in the *i*-th iteration, then record the corresponding *Q*_*ij*_, otherwise, do not record. Note: to facilitate a clear representation of the comparison between the corresponding Pareto surfaces of the algorithm, (the closer the Pareto surface is to the origin, the better the performance of the algorithm), we choose the negative value of the objective function two. At the same time, we still follow this practice within the example study in order to keep the Pareto surface consistent within the article, so as to prevent function confusion.

### 6.2. Particle Decoding

Count the idle equipment models and quantities within each manufacturing enterprise and each subsidiary of the same manufacturing enterprise. At the same time, after simulating the transfer, the Δ*CO*_*aii*′_^*tm*^ of each subsidiary after the transfer of each idle equipment is calculated. Combined with the data to analyze whether there is a pattern in the degree of change of the actual customer demand under this uncertain environment to select the corresponding forecasting method, the actual demand of customers in each cycle is counted by big data and the actual demand of customers in the case where the influence of multiple uncertainties is large. The actual demand of each customer is predicted by the selected forecasting method established ∑_*p*=1_^*P*^*Q*_*pj*_^*tm*^. Combine the profit function of the information generation system of the manufacturing enterprise and the supply and demand matching capability function with the quality of robustness in the worst case. The multiobjective robust optimization model is constructed by combining the constraints. Finally, we combine the NSGA-II-MOPSO algorithm to generate the corresponding scheduling plan and execute the corresponding production plan.

### 6.3. Experimental Parameter Settings

In order to verify the applicability and effectiveness of the improved multiobjective particle swarm genetic algorithm (NSGA-II-MOPSO), this paper applies the algorithm to the multiobjective problem of multisubsidiary and multiequipment production of different scales. The parameters of the simulation example are designed as follows: the maximum value of total demand for the product *Q*_*kmax*_ is 90, 110, 30, 100, and 80; the demand for the product ∑_*i*=1_^*I*^*Q*_*ki*_, *k*=1,…, *K* is randomly generated within [0, *Q*_*kmax*_]; the total number of idle equipment *A* is 20, the output of subsidiary *C*_*i*_ after the transfer occurs *CO*_*i*_^*tmτ*^, the unit price of raw materials *C*_*ij*_^*tmβ*^ of subsidiary *C*_*i*_, the unit price of labor *C*_*ij*_^*tmα*^ of subsidiary *C*_*i*_, the unit price of transportation of equipment a transferred to subsidiary *C*_*i*_ and the unit price of equipment *C*_*aii*′_^*tm*^, *C*_*ai*′_^*tm*^ are randomly generated in [80, 120], [100, 130], [70, 100], [1200, 1400], and [500, 900], respectively. The NSGA-II-MOPSO algorithm uses the following parameters: individual learning coefficient *c*1 *=* 1, population learning coefficient *c*2 = 2, initial weight *ω* = 0.5, crossover probability *P*_*c*_ = 0.5, and genetic probability *P*_*m*_=1/*x*_*num*; among them, *x*_*num* indicates the population size *x*_*num*_=100. The maximum number of iterations *maxIt* = 100, the maximum velocity *V*_max_ is 1; and the algorithm is implemented using MATLAB 2016a programming, and the running environment is a PC with a Pentium IV 2.50 GHz CPU. For comparison, the objective function II is taken as negative and the unit is set to 100,000.

### 6.4. NSGA-II-MOPSO Performance Test

The performance of the NSGA-II-MOPSO algorithm is measured from the quality of the solution and the solution time of the method. Among them, the quality of the solution includes the optimal solution quantity index, uniform distribution index, and spatial extension index. The index of the number of optimal solutions is to add up the number of nondominated solutions obtained by all algorithms to obtain the ratio of the number of nondominated solutions obtained by each algorithm to the summarization.

The uniform distribution metric $\text{Spacing}\left(P\right)=\sqrt{1/\left(N-1\right)\sum_{j=1}^{N}{\left|d_{mean}-d_{j}\right|}^{2}}$ is used to measure the standard deviation of the minimum distance from each solution to the other solutions in the set of nondominated solutions. Among them, *d*_*j*_ indicates the minimum distance from the *j*-th solution in the nondominated solution set to other solutions and *d*_mean_ indicates the mean of all *d*_*j*_.

The spatial extension metric Δ=*d*_*f*_+*d*_*l*_+∑_*i*=1_^*N*−1^|*d*_*i*_ − *d*_mean_|/*d*_*f*_+*d*_*l*_+(*N* − 1)*∗d*_mean_ is used to measure the extensiveness of the obtained set of nondominated solutions. Among them, the parameters *d*_*f*_, *d*_*l*_ are the euclidean distances between the extreme solutions and the boundary solutions of the obtained set of nondominated solutions; *d*_*i*_ is the Euclidean distance between the consecutive solutions of the obtained set of nondominated solutions.

**Figure 10:**
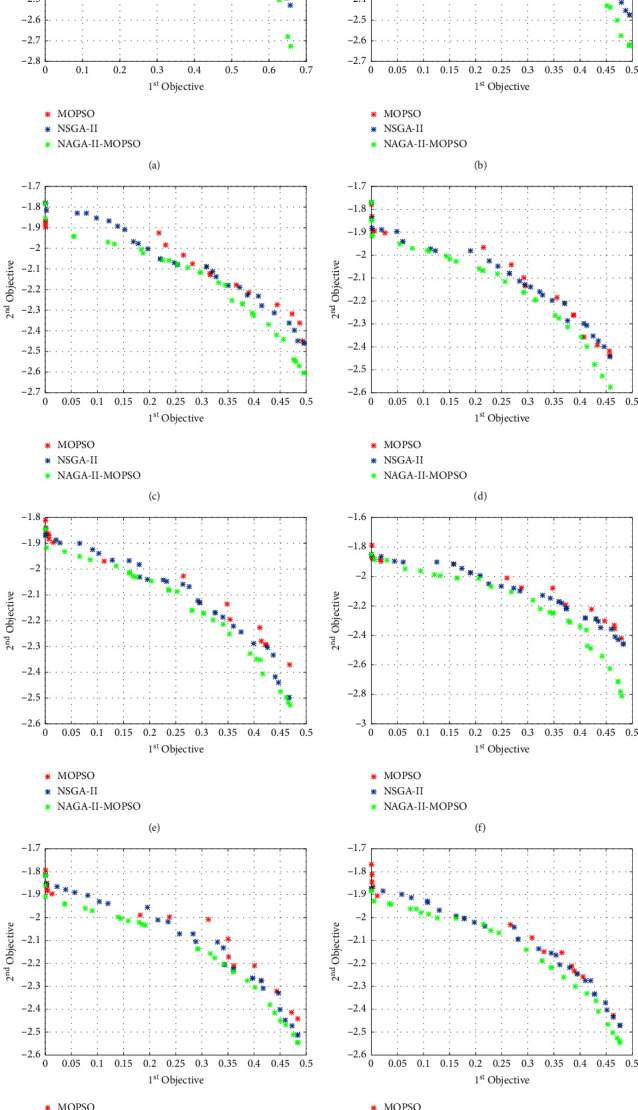
Algorithm testing at different scales: (a) test scale: 100 *∗* 6; (b) test scale: 100 *∗* 9; (c) test scale: 100 *∗* 12; (d) test scale: 100 *∗* 15; (e) test scale: 150 *∗* 6; (f) test scale: 150 *∗* 9; (g) test scale: 150 *∗* 12; and (h) test scale: 150 *∗* 15.

In order to analyze the advantages and disadvantages of MOPSO and NSGA-II in the case of combining this problem, the MOPSO, NSGA-II, and NSGA-II-MOPSO algorithms are compared. Set up the scale of the solution as follows: *a∗*(*b∗c*), among them, *a* denotes the amount of customer demand and (*b∗c*) denotes the product variety and the number of wholesalers. Conduct multiple tests on the scale of the solution. The test results are shown in Figures 10(a)–10(h). Note: the article has images of the Pareto frontier surface in which the horizontal coordinates denote objective function one: minimizing the maximum supply and demand deviation and the vertical coordinates denote objective function two: maximizing corporate profits. Also, for comparison, the objective function II is taken as negative value in US$100,000.

The results are shown in Table 3, and the comparison shows that both MOPSO and NSGA-II have obtained the set of nondominated solutions of the problem. The distribution is uniform and the solution is more detailed and comprehensive. In terms of spatial extension index, NSGA-II-MOPSO is significantly better than the NSGA-II algorithm with a wider search range of nondominated solutions. It is obvious that NSGA-II-MOPSO is significantly better than NSGA-II and MOPSO algorithms in the convergence of Pareto surfaces. The MOPSO algorithm lacks the escape mechanism of local optimum and is easy to fall into the local optimum solution but has higher solution quality. In contrast, NSGA-II greatly improves the global search ability of the algorithm under the perturbation strategies such as the variation and crossover operator. NSGA-II-MOPSO combines the advantages of both algorithms and has significant advantages in the indexes of solution speed, solution quality, solution range, and uniformity of nondominated solutions, which is a convenient and fast fusion algorithm with good solution quality.

### 6.5. Flowchart of Multilayer Game Based Collaborative Optimization Based on Elman System Diagnosis in the Shared Manufacturing Model

**Figure 11:**
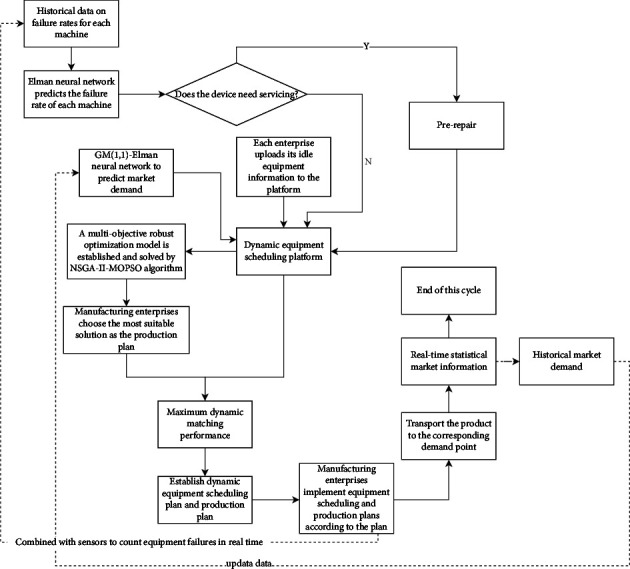
Flowchart of the subsidiary collaborative scheduling system considering dynamic equipment matching efficiency under shared manufacturing.

In summary, the operational process of the collaborative scheduling system of a manufacturing enterprise based on the shared manufacturing model is shown in Figure 11.

  Step 1: The manufacturing enterprise counts the data of historical market demand and puts it into the prediction model and combines the GM(1, 1)-Elman neural network to predict the market demand within this stage. The failure rate of each equipment is predicted and premaintenance treatment is performed before the equipment dispatch occurs
  Step 2: Combine the manufacturing capacity of each subsidiary in the enterprise and build the corresponding multiobjective robust optimization model by decomposing the orders and combine it with NSGA-II-MOPSO algorithm to solve
  Step 3: The manufacturing enterprise selects the most suitable production solution with the actual conditions and the best solution
  Step 4: The manufacturing enterprise establishes a dynamic equipment scheduling platform based on the existing equipment resources and uploads the equipment information and monitors the load capacity of each equipment in real-time through sensors
  Step 5: The manufacturing enterprise establishes the scheduling plan for the equipment based on the maximum dynamic equipment matching efficiency function
  Step 6: Each subsidiary in the manufacturing enterprise dispatches and produces equipment according to the dispatching plan and production plan and transports the products to the corresponding wholesalers
  Step 7: Real-time statistics of the demand information of each wholesaler in the manufacturing enterprise, so as to prepare for the market demand forecast in the next cycle
  Step 8: The terminal of the market statistics in real-time for each parameter value required for the next forecast
  Step 9: End of this cycle

## 7. Model Applications

**Figure 12:**
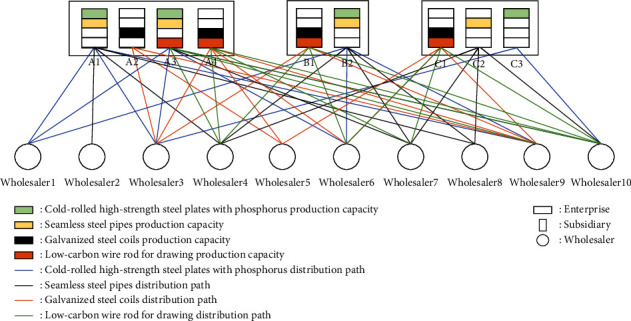
Original system transportation path diagram.

**Figure 13:**
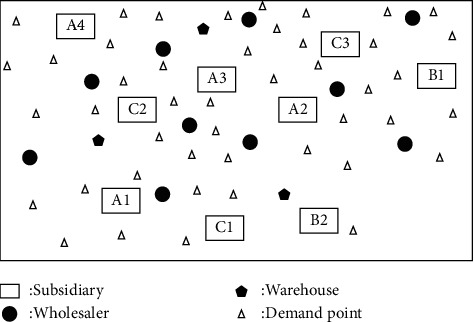
Subsidiaries, warehouses, wholesalers, and demand point distribution plan.

There are three iron and steel enterprises A, B, and C, and there are many subsidiaries in each enterprise. The products produced by the subsidiaries of the enterprise are mainly divided into hot-rolled, cold-rolled, special steel, high-speed wire rod, stainless steel, cast pipe, and other products, and the subsidiaries of each enterprise have independent production capacity. In addition, each enterprise has the independent storage system and logistics system and each subsidiary has the ability to process multiple types of products. Among them, several subsidiaries can independently produce four types of products: cold-rolled plus phosphorus high-strength steel sheets, seamless steel pipes, galvanized steel coils, and low-carbon wire rod for drawing, and some subsidiaries have idle equipment available for dispatch. Now, due to uncertain environmental factors, the actual demand fluctuates significantly. In order to better meet customer demand, each subsidiary within enterprises A, B, and C decides to adopt an intermediary-based sharing platform to share idle equipment in order to enhance its own production capacity. For the sake of differentiation, the subsidiaries with the capability of processing cold-rolled high-strength steel plates with phosphorus are indicated by ◎, the subsidiaries with the capability of processing seamless steel pipes are indicated by ○, the subsidiaries with the capability of processing galvanized steel coils are indicated by ☉, and the subsidiaries with the capability of processing low-carbon wire rod for drawing are indicated by ▽. The main production products of each subsidiary are shown in Table 4. The original system transportation path is shown in Figure 12. The floor plan according to the geographical location of each subsidiary, warehouse, wholesaler, and demand point is shown in Figure 13. Among them, the corresponding subsidiaries of each enterprise are named in the way of “enterprise + subsidiary,” e.g., the subsidiaries of enterprise A are named in order as A1, A2, and A3, respectively.

We combined the GM(1, 1)-Elman neural network to predict the market terminal data and market demand, and aggregated the specific data of each demand point and generated the specific information and predicted values as shown in Table 5.

Combined with the dynamic equipment establishment equation, we calculate the idle equipment that can be replaced by each subsidiary as shown in Table 6. Each subsidiary uploads its own idle equipment information to the shared scheduling platform for equipment sharing among enterprises.

We combined the equation to predict the equipment monitoring values of the existing equipment of each subsidiary in the manufacturing enterprise, as shown in Table 7.

**Figure 14:**
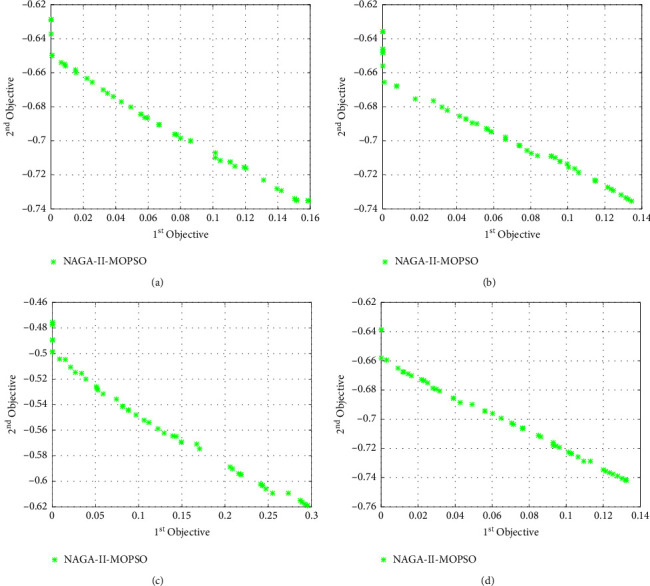
Different products correspond to the Pareto surface. (a) Cold-rolled high-strength steel plates with phosphorus Pareto surface. (b) Seamless steel pipes Pareto surface. (c) Galvanized steel coils Pareto surface. (d) Low-carbon wire rod for drawing Pareto surface.

We solve for the existing market demand and the limitations of each manufacturing enterprise's own production capacity combined with the multiobjective model. The results obtained in combination with the NSGA-II-MOPSO algorithm are shown in Figures 14(a)–14(d).

Combined with the above analysis, the manufacturing enterprise chooses the appropriate production plan according to the Pareto surface. The final production plan and multiobjective function value of each product are shown in Table 8.

**Figure 15:**
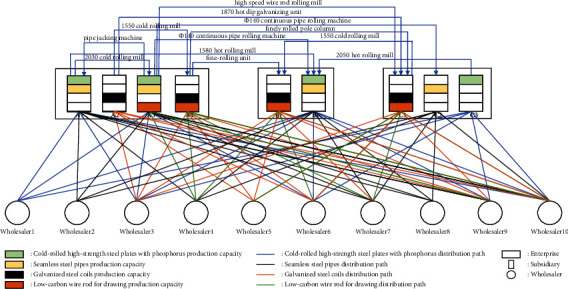
Dynamic equipment scheduling route and production plan.

**Figure 16:**
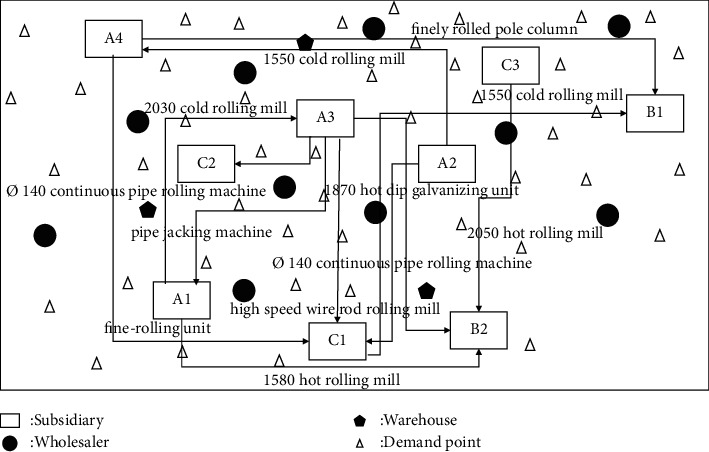
Dynamic equipment scheduling floor plan.

In view of the above results, appropriate equipment is selected from subsidiaries within each enterprise for scheduling. The scheduling routes and plane distribution diagrams of the final dynamic equipment are shown in Figures 15 and 16.

Taking cold-rolled high-strength steel plates with phosphorus as an example, the existing robust solution is calculated to have a cost loss reduction ratio of 24.73% compared to the normal solution in the worst scenario. After case studies, we found that the two-way uncertainty problem consisting of uncertainty in capacity and uncertainty in market demand caused by equipment failure problems in the existing market environment has a great impact on manufacturing enterprises. The dynamic changes in the terminal market demand often make the enterprises face large economic losses or opportunity cost losses. During the production process, the occurrence of equipment failure problems and the uncertainty of maintenance effectiveness cause uncertainty in production output. Combining model predictive control methods will greatly alleviate this problem and increase the robustness and stability of the system during operation.

## 8. Conclusion and Development Suggestions

In the field of manufacturing, the multilayer game optimization method based on Elman neural network system diagnosis proposed in this paper is closely combined with the analysis of market terminal big data, so that the system is always in a continuous dynamic optimization state. At the same time, we propose a robust optimization method to maintain good production characteristics in the worst-case scenario by closely integrating the dynamic market demand and the uncertain changes of manufacturing capacity of manufacturing enterprises. We combine the Elman neural network with the model predictive control method to further improve the system robustness and combine the NSGA-II-MOPSO algorithm to achieve the production plan optimization and decision-making. In the actual production process, the model predictive control method mentioned in this paper is closely combined with the terminal data analysis, and a large number of sensor devices and technologies are required to realize real-time monitoring of products and equipment. The intelligence, wireless, miniaturization, and integration of sensors will become one of the keys to the development of future manufacturing technology. Based on the method proposed in this paper, the manufacturing enterprise can adjust the production planning arrangement to realize the dynamic forecasting of the production process, and further improve the enterprise benefit. For the actual production of the enterprise, we propose the following suggestions:

- Realize a high degree of intelligence in the production process and real-time collection and monitoring of production information in the production process.
- Realize the intelligent optimal allocation of resources and realize the sharing of information and resources among enterprises through mutual circulation. The intelligent and networked manufacturing produced by the integration of information technology and manufacturing technology can allocate resources across regions, breaking through the original localized production boundaries.
- Realize highly intelligent and personalized products. Enterprises can realize self-monitoring, recording, feedback, and remote control functions by setting sensors, controllers, memory, and other technologies on built-in products.

We believe that this model method can be applied to practical problems where multiple dynamic factors affect the system, such as traffic planning, hospital surgery scheduling, and bus route planning.

## Figures and Tables

**Table 1:** Grade comparison table.

Fuzzy accuracy level	Mean squared error ratio *C*
Level 1 (good)	C ≤ 0.35
Level 2 (qualified)	0.35 < C ≤ 0.5
Level 3 (barely)	0.5 < C ≤ 0.65
Level 4 (unqualified)	0.65 < C

**Table 2:** Demand probability table.

Demand	*d*_1_	*d*_2_	*d*_3_	…	*d*_*p*_
Probability	*P*(*d*_1_)	*P*(*d*_2_)	*P*(*d*_3_)	…	*P*(*d*_*p*_)
*P*(*D*)	1	1 − ∑_*p*=1_^1^*p*(*d*_*p*_)	1 − ∑_*p*=1_^2^*p*(*d*_*p*_)	…	*P*(*d*_*p*_)

**Table 3:** Algorithm comparison results.

Test scale	Optimal solution quantity index			Spacing (*p*)			Spatial extension indicator			Time (*t*)		
	Multiobjective particle swarm optimization algorithm (MOPSO)	Nondominated sorted genetic algorithm-II (NSGA-II)	NSGA-II-MOPSO	MOPSO	NSGA-II	NSGA-II-MOPSO	MOPSO	NSGA-II	NSGA-II-MOPSO	MOPSO	NSGA-II	NSGA-II-MOPSO
100 *∗* 6	0.200	0.400	0.400	0.010	0.350	0.040	1.812	0.827	0.778	64.378	0.732	6.289
100 *∗* 9	0.231	0.340	0.429	0.025	0.104	0.040	1.511	0.670	0.724	79.273	0.912	6.720
100 *∗* 12	0.211	0.350	0.439	0.014	0.105	0.040	1.794	0.703	0.849	85.543	1.351	7.680
100 *∗* 15	0.221	0.362	0.417	0.006	0.104	0.013	1.588	0.591	0.618	87.495	1.329	10.936
150 *∗* 6	0.221	0.378	0.401	0.034	0.096	0.029	1.716	0.731	0.772	107.980	2.324	12.026
150 *∗* 9	0.241	0.386	0.373	0.005	0.120	0.041	1.594	0.714	0.799	101.720	2.225	12.796
150 *∗* 12	0.221	0.345	0.434	0.013	0.105	0.001	1.775	0.696	0.647	105.101	3.273	11.384
150 *∗* 15	0.200	0.390	0.410	0.027	0.081	0.014	1.806	0.531	0.647	153.082	3.337	12.472

**Table 4:** Table of main production products of subsidiaries.

Product categories	Products	Subsidiaries								
		A1	A2	A3	A4	B1	B2	C1	C2	C3
Cold-rolled products	Cold-rolled high-strength steel plates with phosphorus	◎		◎			◎			◎
Casting products	Seamless steel pipes	○		○			○		○	
Cold-rolled products	Galvanized steel coils		☉		☉	☉		☉		
High-speed wire products	Low-carbon wire rod for drawing			▽	▽	▽		▽		

**Table 5:** Statistical table of market terminal data.

Wholesaler	Customer purchasing power				Product market share				Market terminal consumption ($)				Customer purchase frequency			
	Cold-rolled high-strength steel plates with phosphorus	Seamless steel pipes	Galvanized steel coils	Low-carbon wire rod for drawing	Cold-rolled high-strength steel plates with phosphorus	Seamless steel pipes	Galvanized steel coils	Low-carbon wire rod for drawing	Cold-rolled high-strength steel plates with phosphorus	Seamless steel pipes	Galvanized steel coils	Low-carbon wire rod for drawing	Cold-rolled high-strength steel plates with phosphorus	Seamless steel pipes	Galvanized steel coils	Low-carbon wire rod for drawing
1	0.93	0	0	0	0.48	0	0	0	201560	0	0	0	0.86	0	0	0
2	0	0.8	0	0	0	0.69	0	0	0	159820	0	0	0	0.78	0	0
3	0.96	0	0.88	0	0.79	0	0.83	0	175600	0	172300	0	0.79	0	0.83	0
4	0	0.79	0	0.86	0	0.86	0	0.76	0	1952000	0	187500	0	0.79	0	0.84
5	0	0	0.95	0	0	0	0.84	0	0	0	186245	0	0	0	0.85	0
6	0.87	0	0.89	0.94	0.69	0	0.59	0.69	183000	0	176985	169850	0.91	0	0.93	0.87
7	0	0.83	0.91	0.89	0	0.93	0.86	0.92	0	186396	196452	175200	0	0.86	0.76	0.59
8	0.89	0.85	0	0	0	0.46	0	0	0	204598	0	0	0	0.94	0	0
9	0.86	0	0.89	0.96	0.94	0	0.75	0.93	195200	0	192417	169853	0.76	0	0.86	0.64
10	0.93	0.87	0	0.89	0.85	0.85	0	0.85	172500	186592	0	185420	0.79	0.86	0	0.85

**Table 6:** List of idle equipment of each subsidiary.

Subsidiary	Dynamic equipment
A1, B2, and C3	2030 cold rolling mill
A1, C3	1580 hot rolling mill
A3, B2	2050 hot rolling mill
A3	Pipe jacking machine
A1, A3	Φ140 continuous pipe rolling machine
A2, C1	1550 cold rolling mill
A2	1870 hot dip galvanizing unit
A3, A4, and B1	High-speed wire rod rolling mill
A3, A4	Fine-rolling unit

**Table 7:** Monitoring table of equipment corresponding to sensors.

Factor		Dynamic equipment								
		2030 cold rolling mill	1580 hot rolling mill	2050 hot rolling mill	Pipe jacking machine	Φ140 continuous pipe rolling machine	1550 cold rolling mill	1870 hot dip galvanizing unit	High-speed wire rod rolling mill	Fine-rolling unit
Degree of equipment depreciation		0.21	0.15	0.13	0.19	0.16	0.24	0.13	0.17	0.2

Equipment load	1	0.79	0.75	0.68	0.79	0.58	0.82	0.76	0.92	0.76
	2	0.86	0.84	0.58	0.68	0.67	0.96	0.84	0.76	0.92
	3	0.69	0.51	0.67	0.76	0.84	0.79	0.68	0.79	0.95
	4	0.87	0.68	0.79	0.84	0.83	0.80	0.94	0.68	0.76

Worker proficiency	1	0.95	0.93	0.86	0.89	0.84	0.79	0.82	0.98	0.96
	2	0.93	0.98	0.83	0.86	0.95	0.93	0.94	0.89	0.97
	3	0.96	0.89	0.83	0.89	0.96	0.86	0.96	0.98	0.99
	4	0.87	0.86	0.94	0.98	0.97	0.95	0.87	0.98	0.86

Historical number of repairs (frequency)		1	3	2	1	2	3	2	2	1

**Table 8:** Production scheme table.

Product	Transportation volume (*t*)	Subsidiary					
		A1	A3	B2	C3	Objective function I	Objective function II ($100,000)
	Wholesaler
Cold-rolled high-strength steel plates with phosphorus	1	10.938	28.480	22.171	0.073	0.086	−0.700
	3	18.045	21.615	32.954	18.711		
	6	0.384	4.926	19.418	25.853		
	9	0.017	25.712	12.782	36.094		
	10	11.199	16.882	24.307	16.981		

Product		A1	A3	B2	C2		
Seamless steel pipes	2	22.517	12.519	23.081	3.061	0.072	−0.704
	4	5.386	8.177	43.543	23.850		
	7	5.223	0.350	35.563	10.445		
	8	30.775	29.936	5.488	10.994		
	10	28.853	3.812	6.507	39.232		

Product		A2	A4	B1	C2		
Galvanized steel coils	3	2.198	26.405	23.824	6.761	0.171	−0.575
	5	9.252	6.861	32.987	11.207		
	6	11.890	9.930	10.943	22.287		
	7	6.047	30.082	3.634	20.762		
	9	18.289	0.662	12.327	21.644		

Product		A3	A4	B1	C1		
Low-carbon wire rod for drawing	4	20.035	13.885	15.093	5.518	0.066	−0.698
	6	15.639	5.270	36.833	23.505		
	7	1.532	18.975	3.399	25.372		
	9	3.890	34.710	30.114	10.277		
	10	28.198	16.547	5.617	33.251		

## Data Availability

The data used to support the findings of this study are available from the corresponding author upon request.
